# Petrological, geochemical (major, trace, and rare earth elements), and U–Pb zircon data of the Tamatán Group, NE Mexico

**DOI:** 10.1016/j.dib.2021.106846

**Published:** 2021-02-17

**Authors:** Juan Moisés Casas-Peña, Juan Alonso Ramírez-Fernández, Fernando Velasco-Tapia, Eduardo Alejandro Alemán-Gallardo, Carita Augustsson, Bodo Weber, Dirk Frei, Uwe Jenchen

**Affiliations:** a Programa de Posgrado de la Facultad de Ciencias de la Tierra, Universidad Autónoma de Nuevo León, Carr. Cerro Prieto Km 8, Ex. Hacienda de Guadalupe, Linares, NL 67700, Mexico; b Universidad Autónoma de Nuevo León (UANL), Facultad de Ciencias de la Tierra, Carr. Cerro Prieto Km 8, Ex. Hacienda de Guadalupe, Linares, NL 67700, Mexico; c Department of Energy Resources, Universitetet i Stavanger, Faculty of Science and Technology, 4036 Stavanger, Norway; d Departamento de Geología, Centro de Investigación Científica y de Educación Superior de Ensenada (CICESE), Ensenada, Mexico; e Department of Earth Sciences, University of the Western Cape, Cape Town, South Africa

**Keywords:** Gondwana, Huizachal peregrina anticlinorium, Paleozoic, Sedimentary petrology, Geochemistry, U-Pb geochronology

## Abstract

From samples of the Paleozoic Tamatán Group (Huizachal–Peregrina Anticlinorium, Tamaulipas, Mexico), petrographic (qualitative and modal) and geochemical analyses (major, trace, and rare earth elements) were conducted. The first U–Pb geochronological data on detrital zircons of the Tamatán Group were generated using four samples. The data presented here contains a broad overview of photomicrography, recalculated modal point-count data, raw geochemical data, and simple statistics of selected geochemical parameters. The data presented in this article are interpreted and discussed in the research article titled “Provenance and tectonic setting of the Tamatán Paleozoic sequence, NE Mexico: Implications for the closure of the Rheic Ocean at the northwestern part of Gondwana” [1].

## Specifications Table

**Table:** 

Subject	Earth Sciences
Specific subject area	Petrology, geochemistry, U–Pb geochronology
Type of data	Microscopy and CL images, tables, figures, and graphs
How data was acquired	Seventy-three samples were pulverized and analyzed using ICP-ES (oxides, Ba, Ni, and Sc) and ICP-MS (trace and rare earth elements) at ACME Laboratories, Vancouver, Canada. The U–Pb analysis followed standard procedures and was carried out at the Geology Department at Centro de Investigación Científica y de Educación Superior de Ensenada Baja California. U–Pb data of four samples were analyzed in detrital zircon grains using laser ablation multicollector inductively coupled plasma mass spectrometry at the Central Analytical Facilities, Stellenbosch University, South Africa.
Data format	Raw (photos, geochemical, and U–PB data), analyzed, processed, and filtered data.
Parameters for data collection	Representative samples for each geological unit were collected in the Peregrina and Caballeros canyons. Thin sections were prepared, point-counted, and photographed.
Description of data collection	Petrological, geochemical, and U–Th–Pb analysis of the sedimentary rocks of the Tamatán Group.
Data source location	Huizachal–Peregrina Anticlinorium, Tamaulipas, Mexico. Caballeros Canyon (23°47.7′–23°49.2′N, and 99°16.8′–99°18′W) Peregrina Canyon (23°46.2′–23°47 °.4 N and 99°14.7–99°16.5W
Data accessibility	Data available within this article and in https://data.mendeley.com/datasets/wbzzy6hcgj/1.
Related research article	Casas-Peña, M., Ramírez-Fernández, J.A., Velasco-Tapia, F., Alemán-Gallardo, E.A., Augustsson, C., Weber, B., Frei, D. & Jenchen, U. (en prep): Provenance and tectonic setting of the Paleozoic Tamatán Group, NE Mexico: Implications for the closure of the Rheic Ocean – Gondwana Research 91 (2021) xxx. – [doi: https://doi.org/10.1016/j.gr.2020.12.012] [1]

## Value of the Data

• First comprehensive data set of selected data from the Paleozoic Tamatán Group, NE México, including petrográphical, geochemical, and Uran/Lead data of all described formations.
• Important information to complete an integrated geological model for the Northwestern Margin of Gondwana and adjacent areas.
• Raw-, and processed data to work with studies of tectonic activity, weathering, and provenance of the Paleozoic sequences of NE Mexico and sedimentary rocks of comparable tectonic settings. This set also includes data which are not used in the related research article to be useful for further investigations.

## 1 This Article

This article provides selected data from 105 samples (for the complete data set, please consult [2]). From 70 samples, photomicrographs were taken and point-counted and modal analyses on recalculated petrographic parameters were provided. Geochemical analyses (major, trace, and rare earth elements [REE]) of 73 samples were conducted. Four samples for U–Pb geochronological zircon analyses were made. The sample location is given with the geographical and UTM coordinates of each sample. Each sample is located on a geological map. The petrographic and geochemical data are presented as raw data and displayed as a simple statistic of the selected petrography and geochemical parameters, respectively. Additionally, outcrop photographs are provided.

**Fig. 1:**
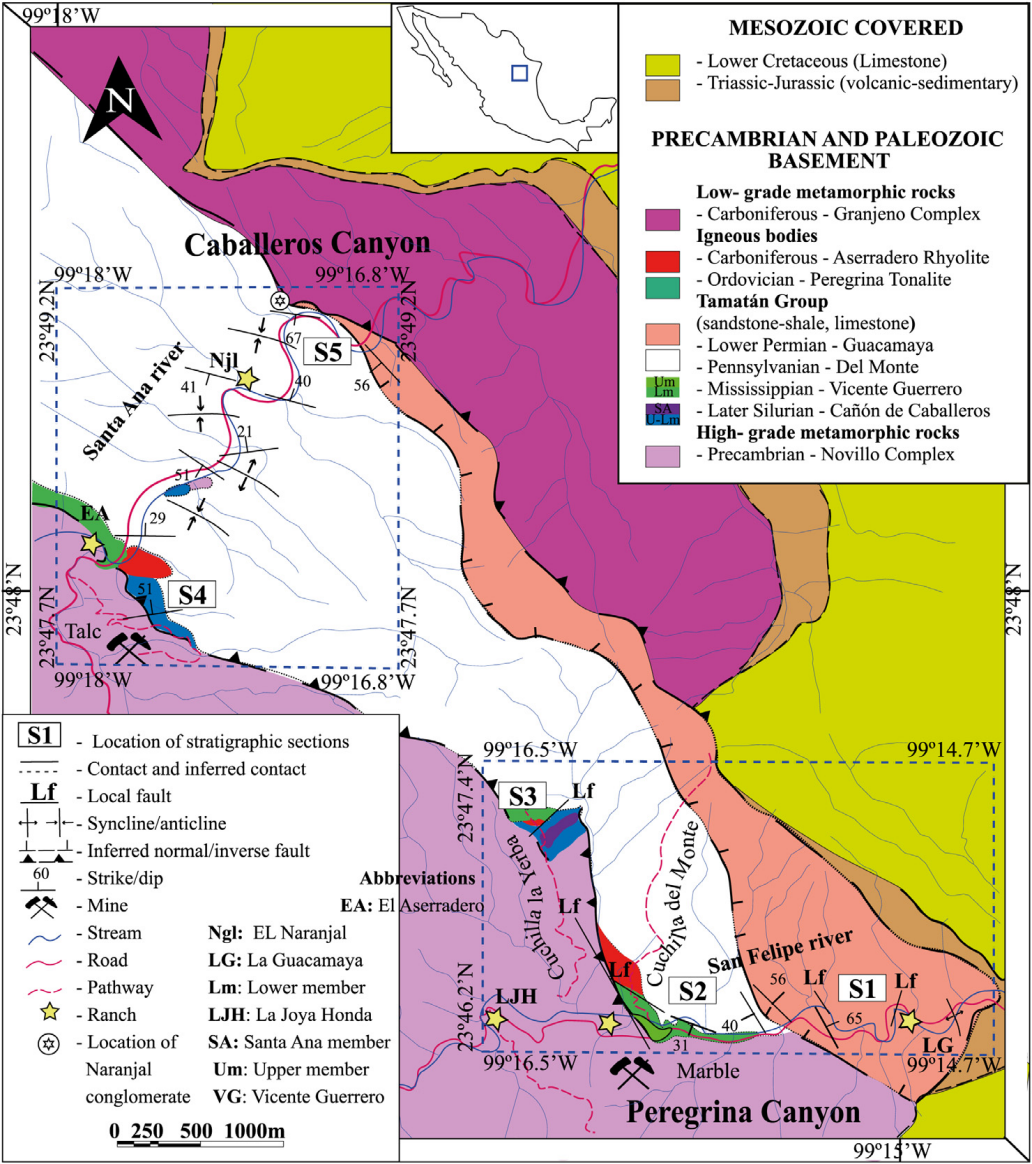
Geological maps of the sampling area (modified from [17,18], and [20]).

**Fig. 2:**
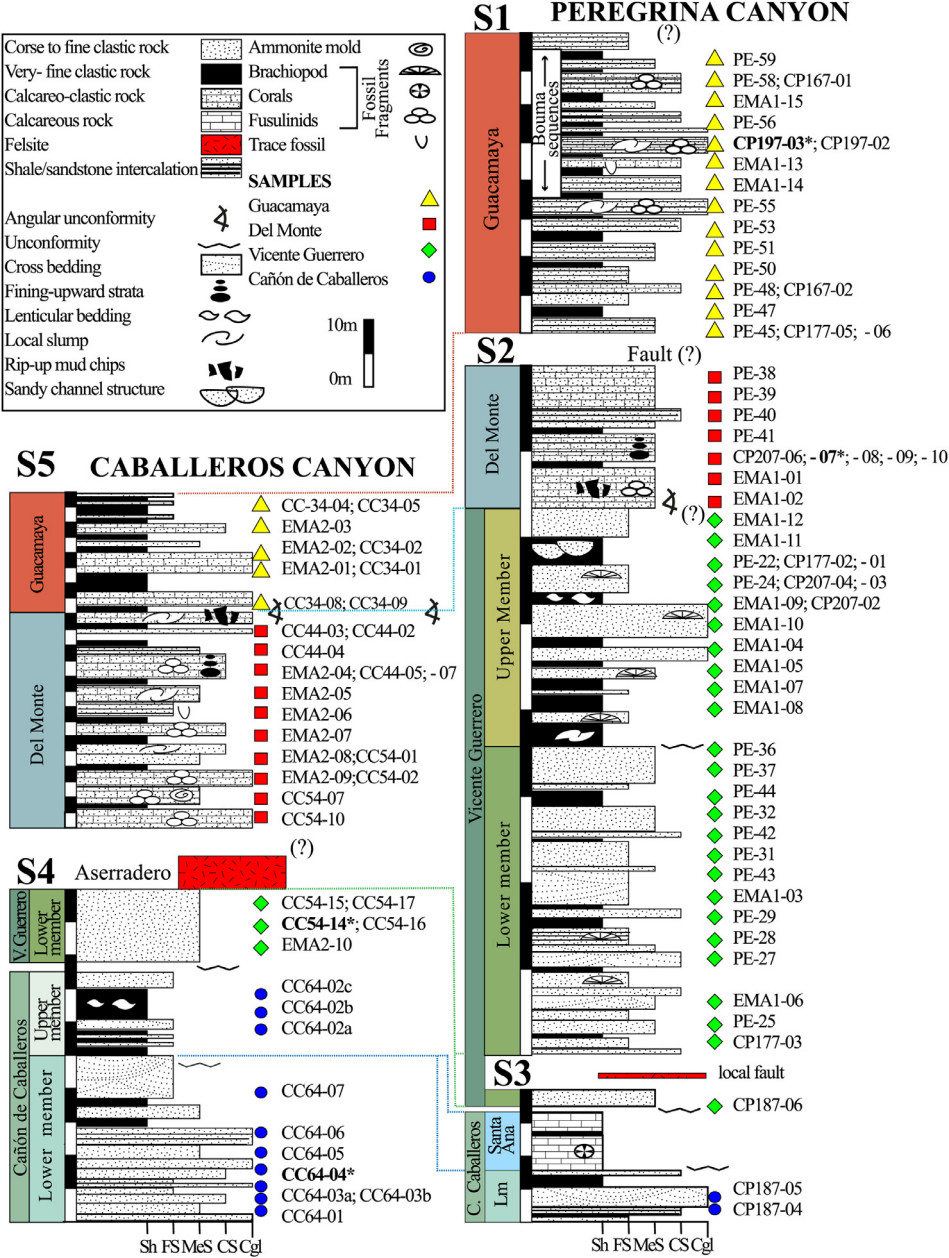
Stratigraphic column and stratigraphic position of samples (modified after [1] and [17]).

**Fig. 3:**
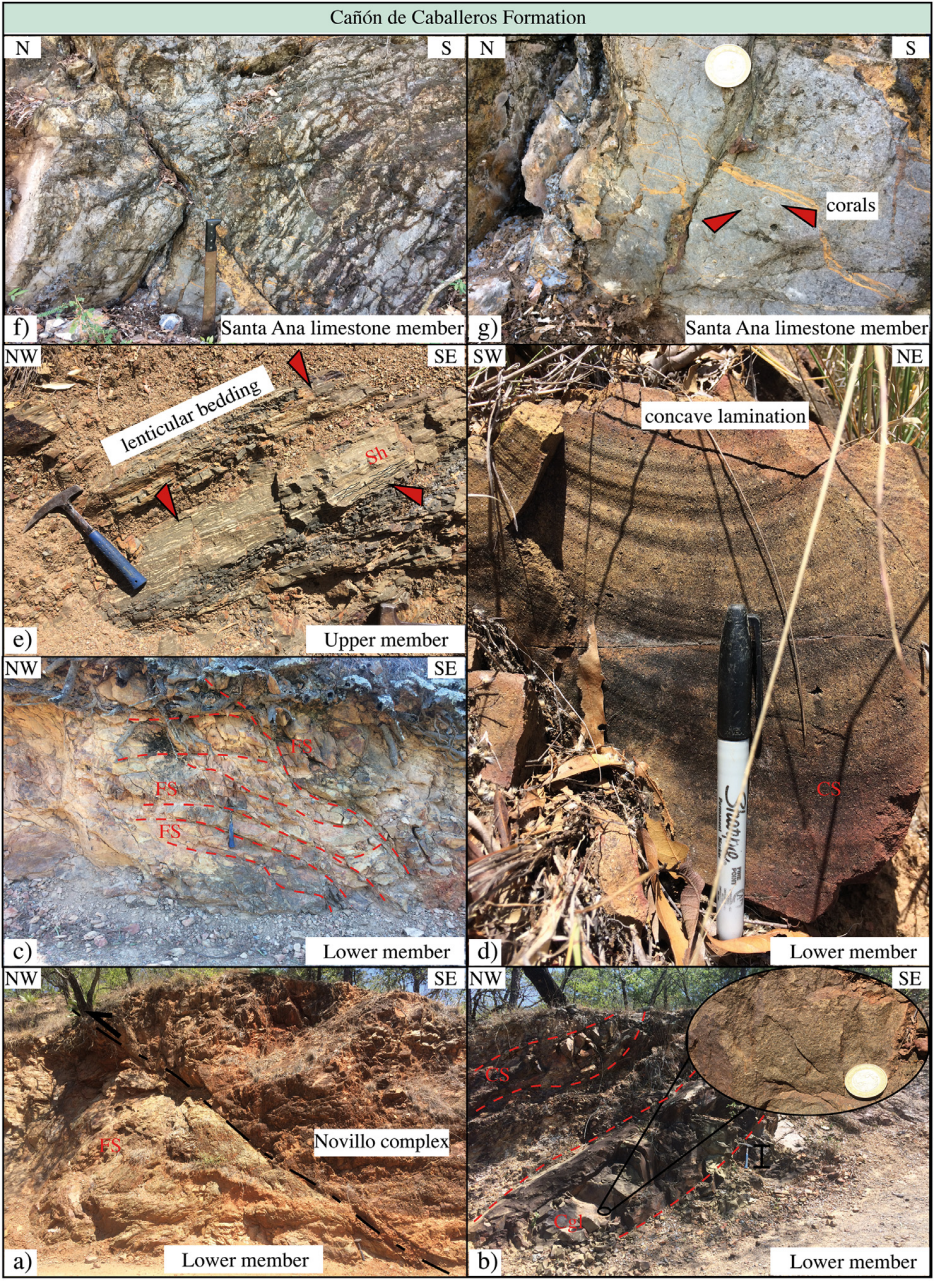
Outcrop photographs from the Cañón de Caballeros Formation (Wenlock to Lower Devonian).

**Fig. 4:**
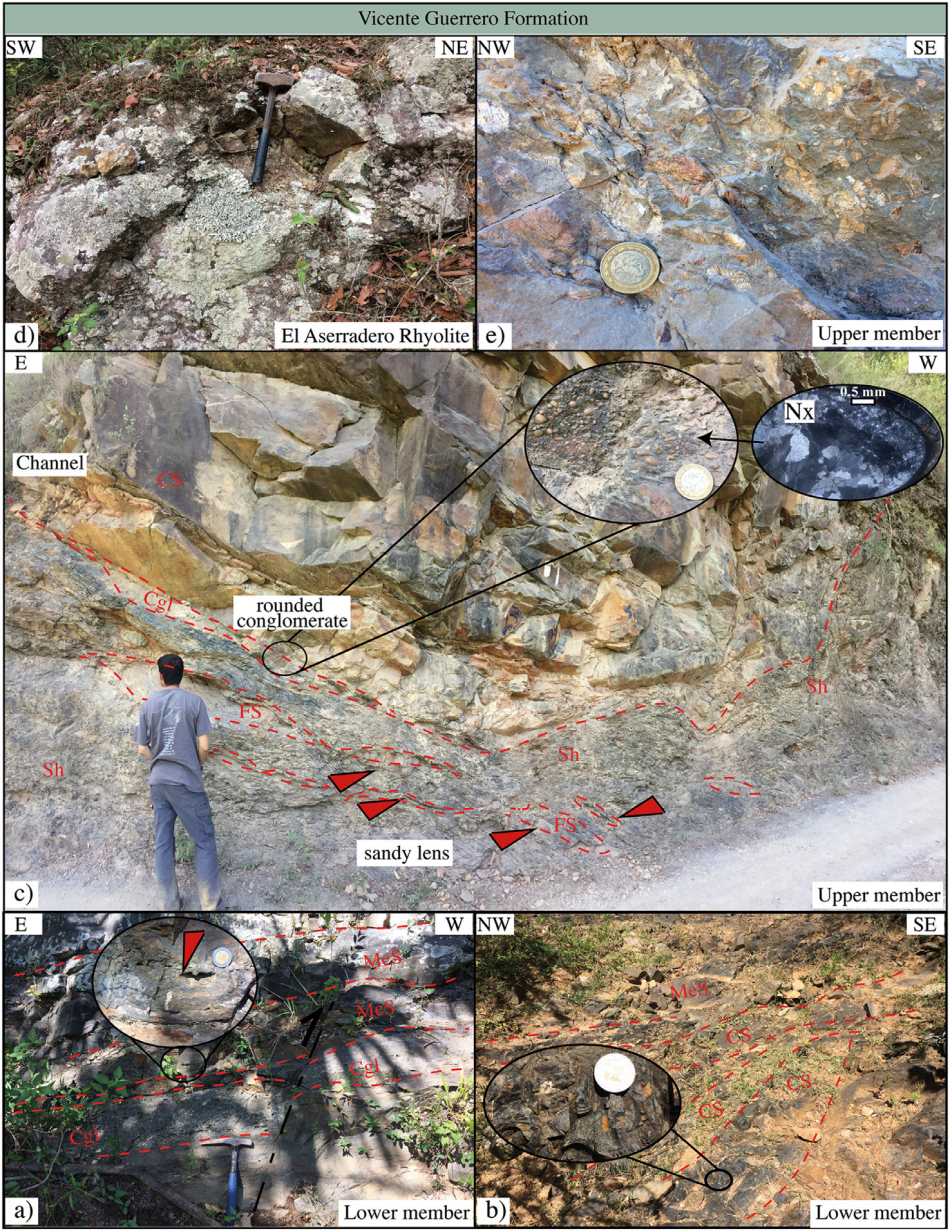
Outcrop photographs from the Vicente Guerrero Formation (Lower Missisipian).

**Fig. 5:**
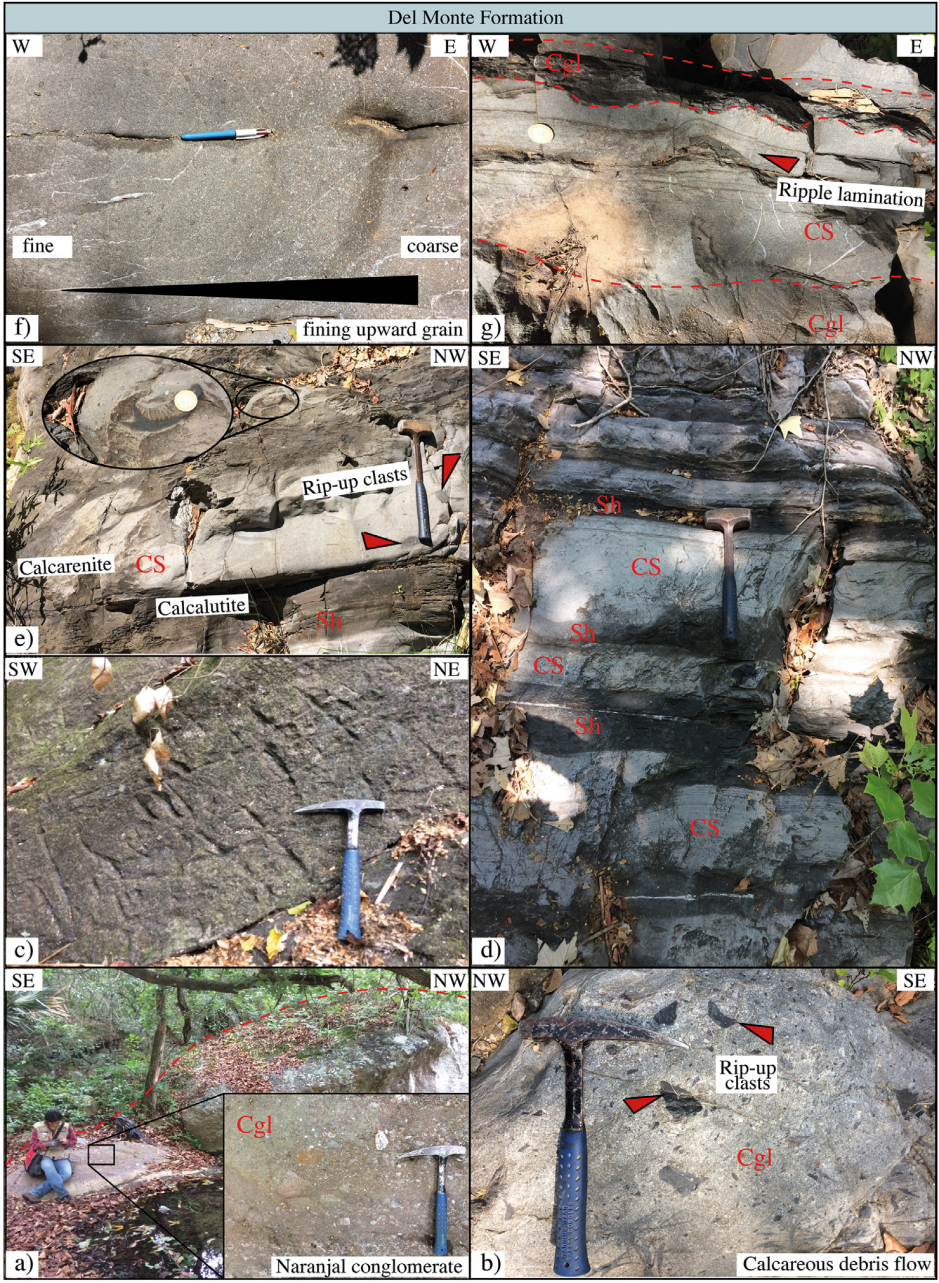
Outcrop photographs from the Del Monte Formation (Lower Pennsylvanian).

**Fig. 6:**
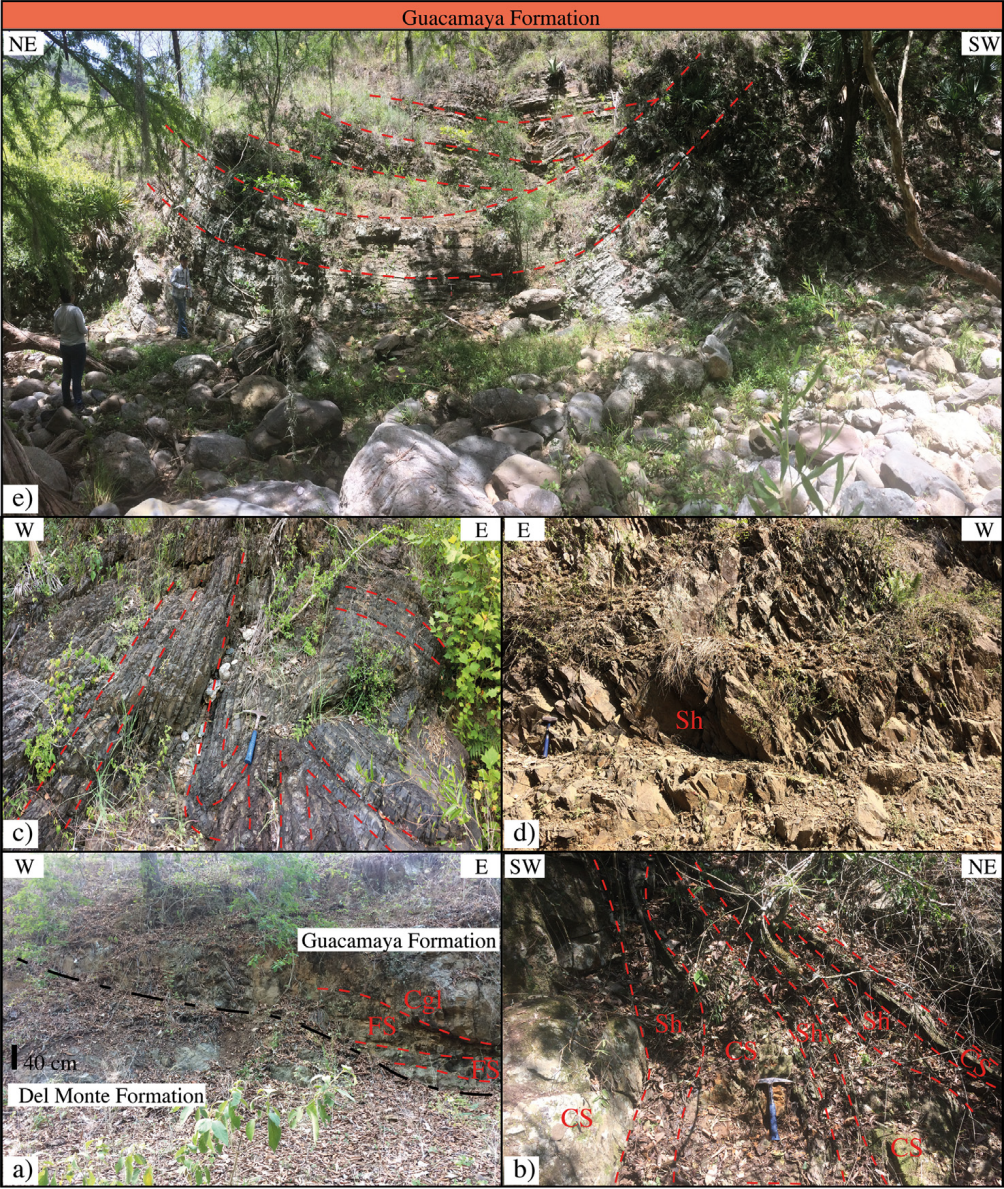
Outcrop photographs of the Guacamaya Formation I (Lower Permian).

**Fig. 7:**
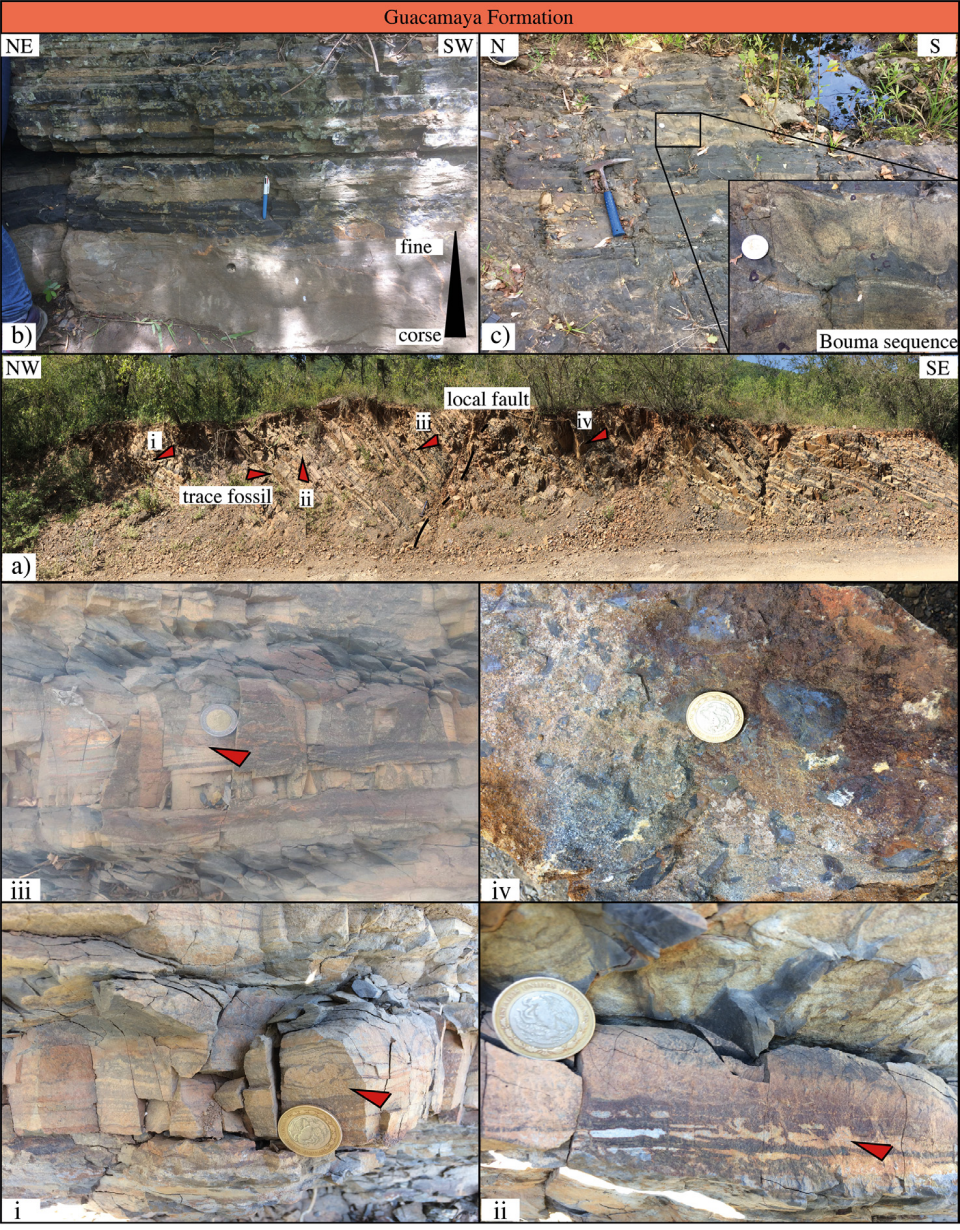
Outcrop photographs from the Guacamaya Formation II (Lower Permian).

**Fig. 8:**
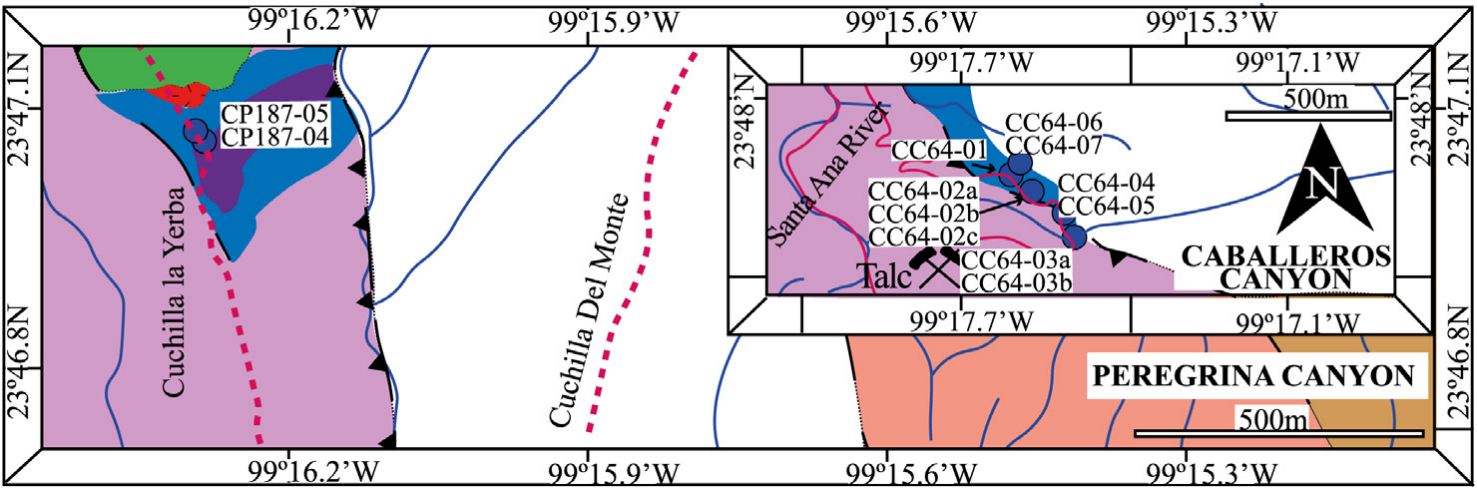
Sample locations of the Cañón de Caballeros formation (Upper Silurian-Lower Devonian).

**Fig. 9:**
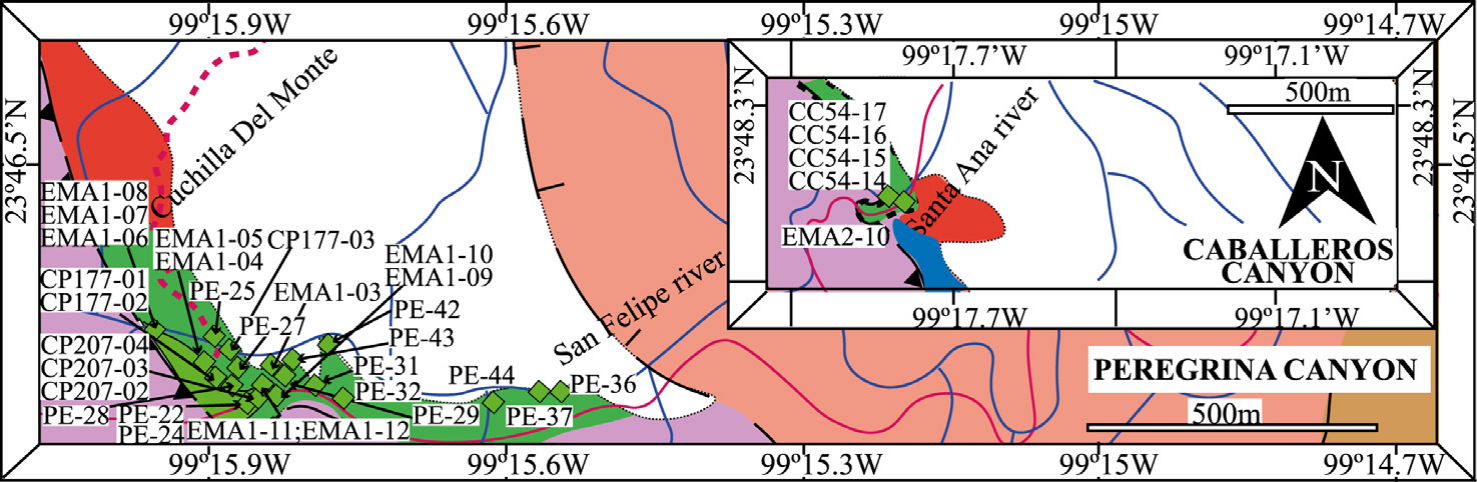
Sample locations of the Vicente Guerrero formation (Mississippian).

**Fig. 10:**
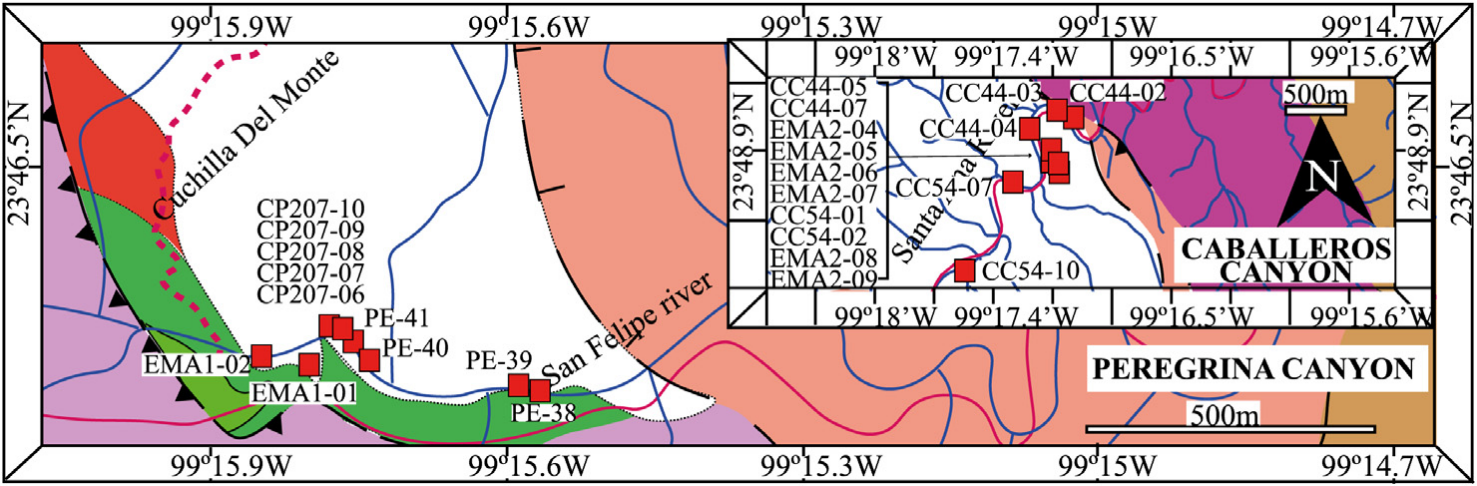
Sample locations of the Del Monte formation (Pennsylvanian).

**Fig. 11:**
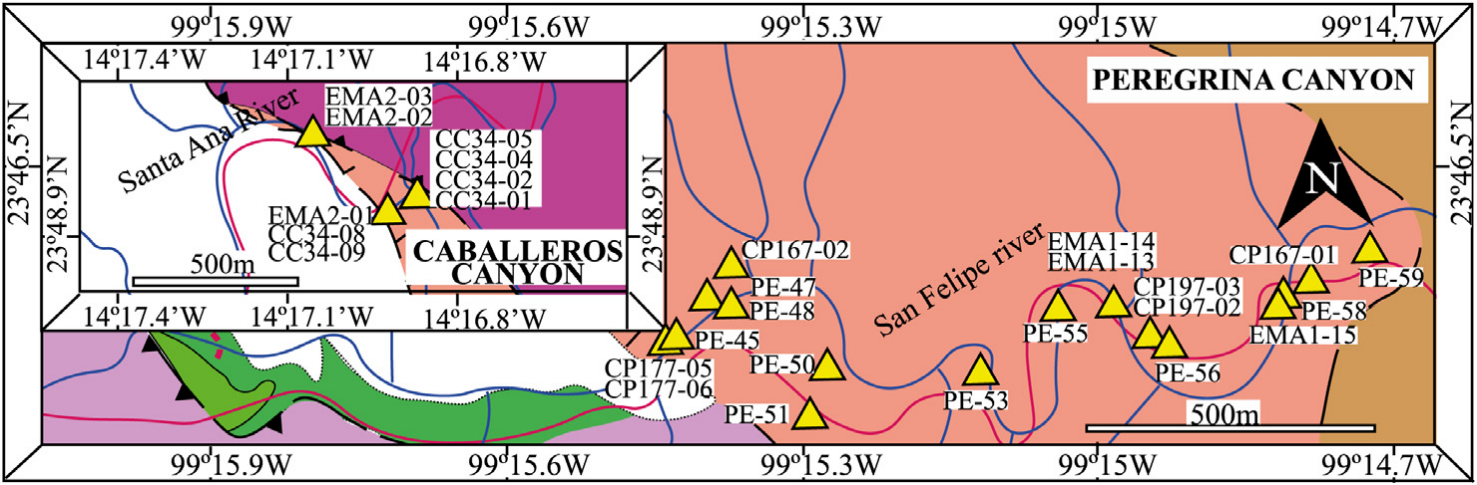
Sample locations of the Guacamaya formation (Permian).

**Fig. 12:**
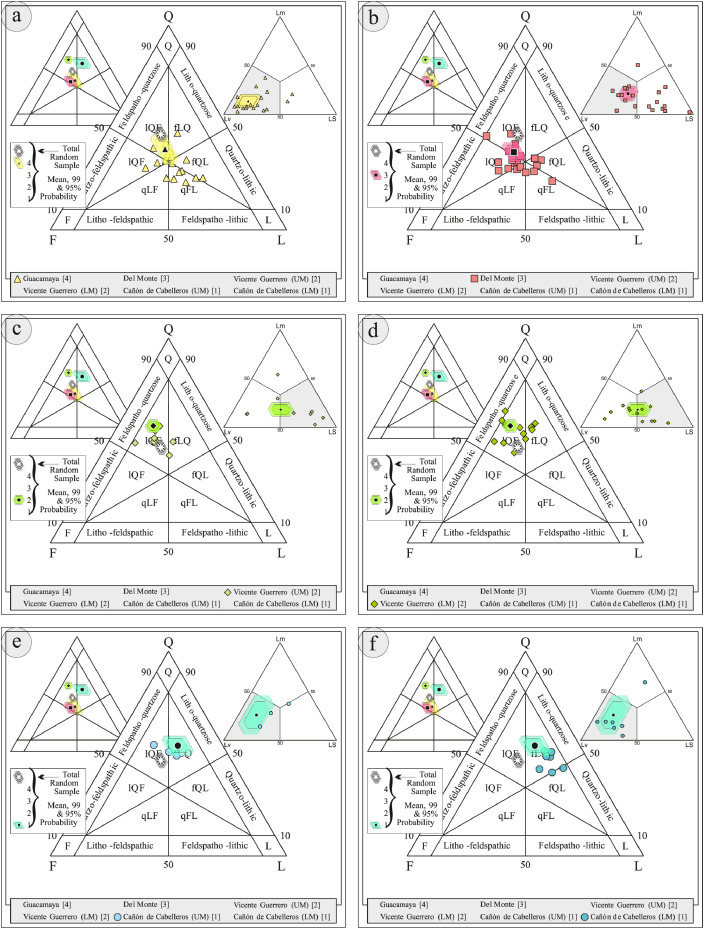
Petrographic modal analysis of Tamatán Group sandstones: Q–F–L after [8].

**Fig. 13:**
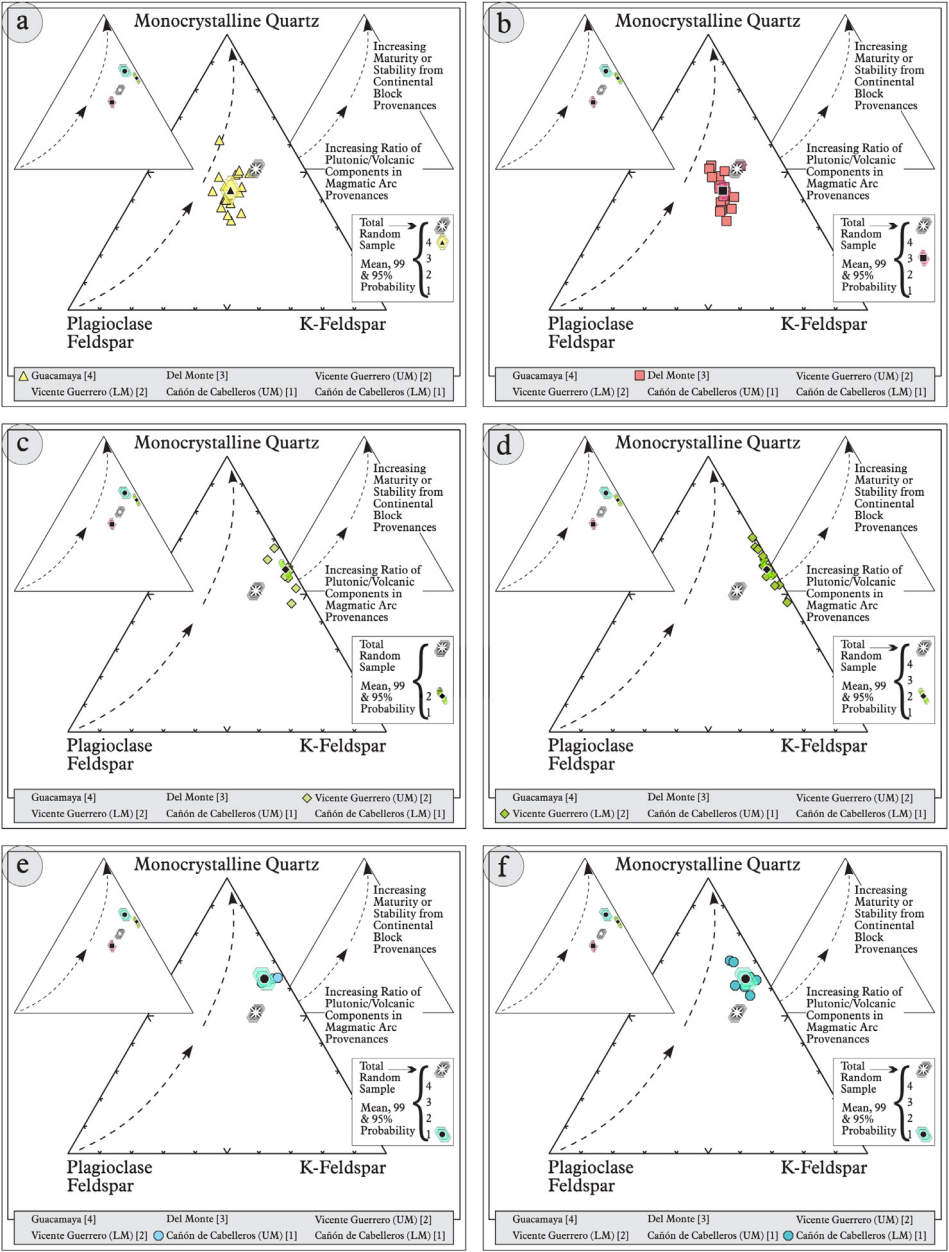
Petrographic modal analysis of Tamatán Group sandstones: Qm–P–K after [6].

**Fig. 14:**
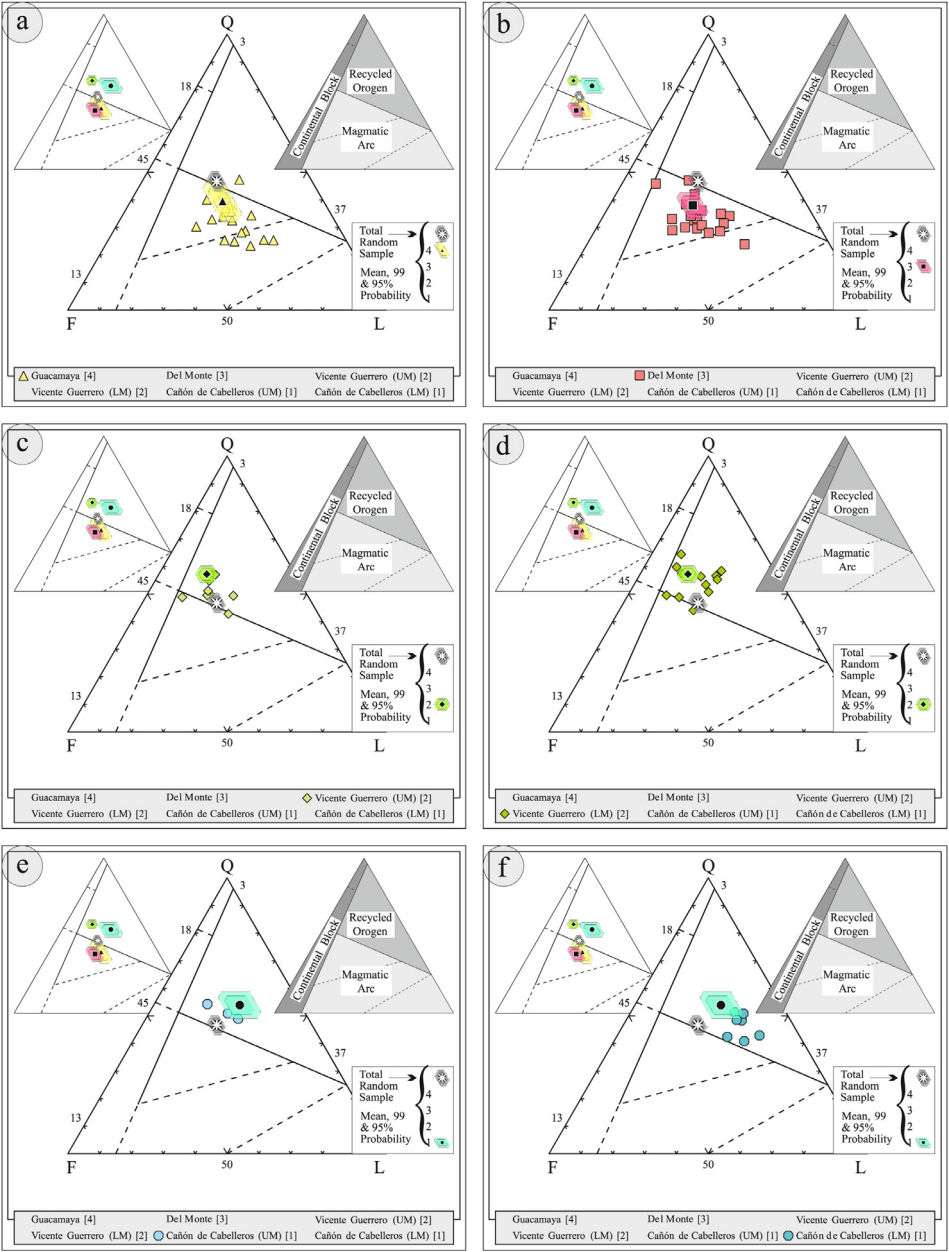
Petrographic modal analysis of Tamatán Group sandstones: Q–F–L after [4].

**Fig. 15:**
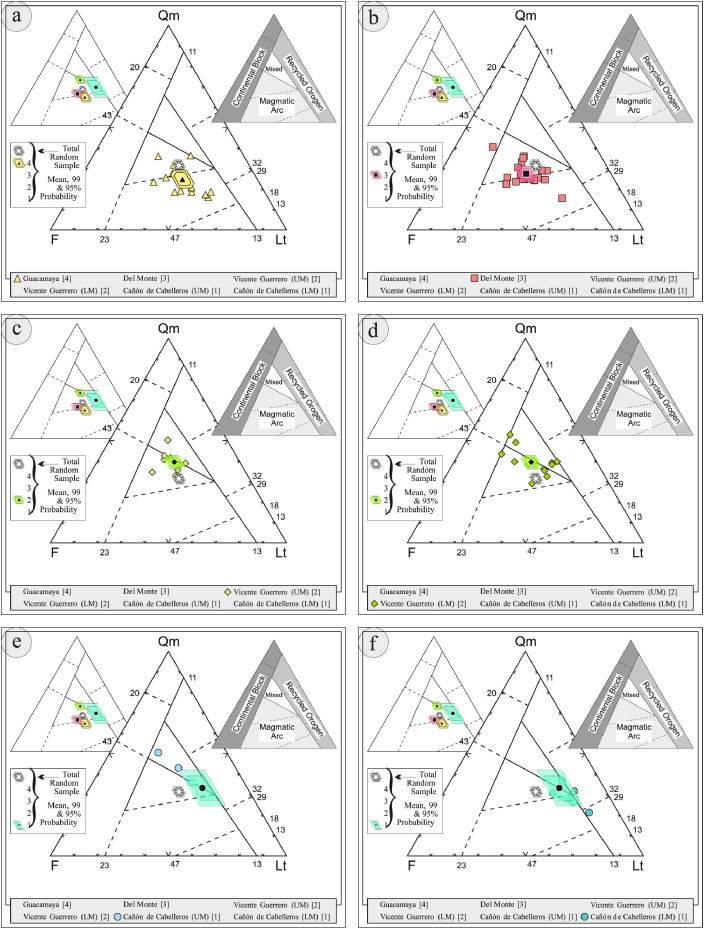
Petrographic modal analysis of Tamatán Group sandstones: Qm–F–Lt after [9].

**Fig. 16:**
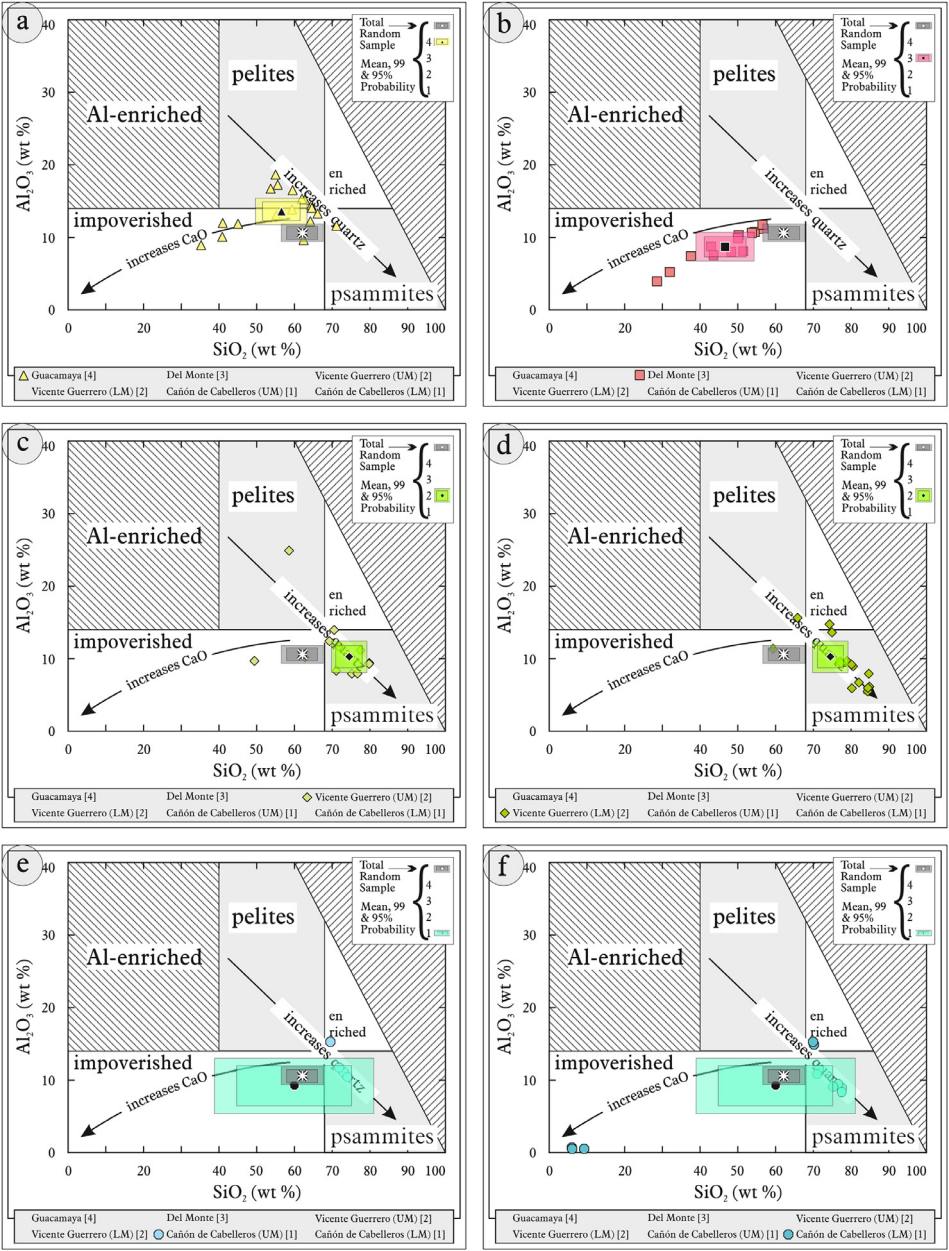
Geochemical sediment classification of the Tamatán Group: SiO_2_–Al_2_O_3_ diagram after [10].

**Fig. 17:**
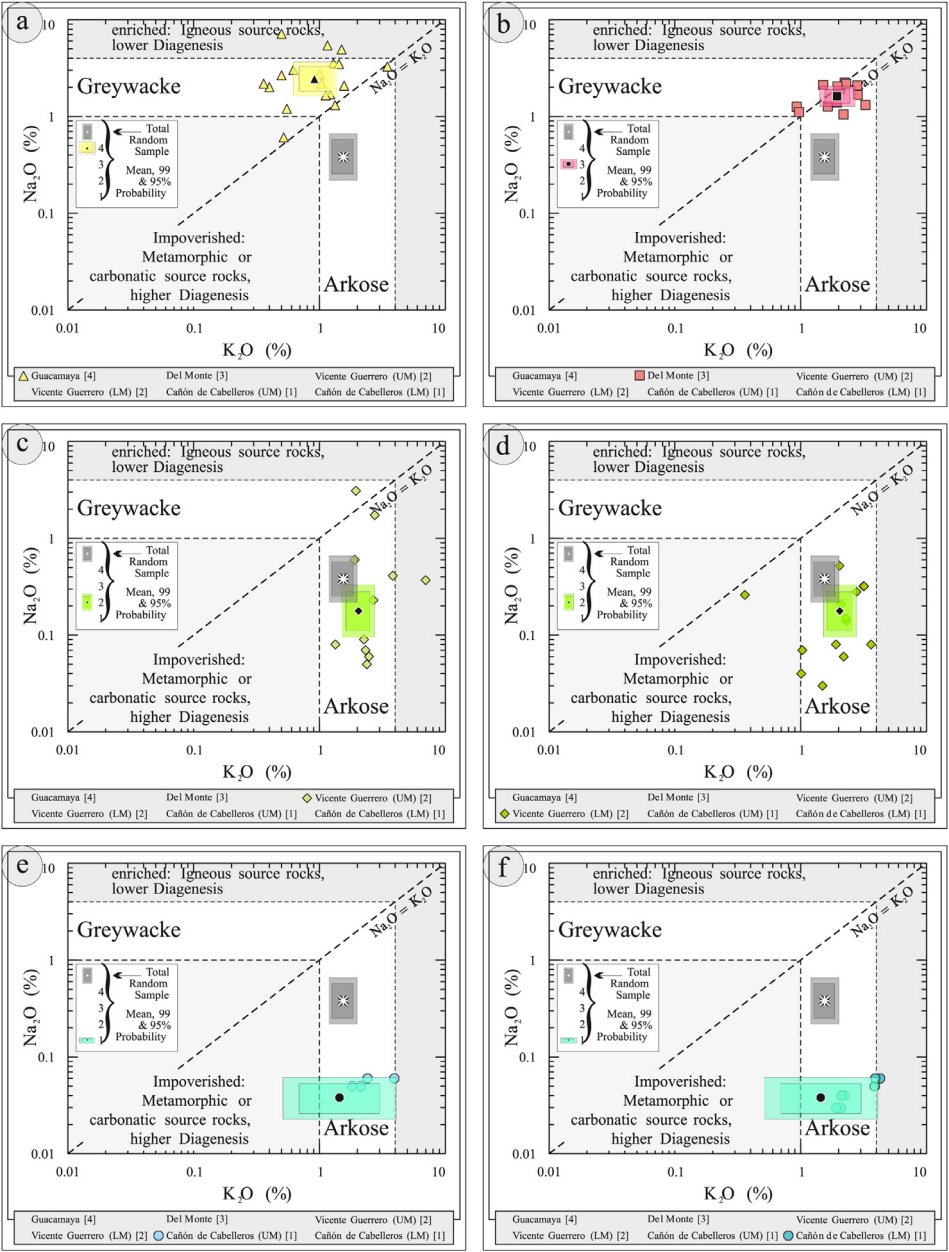
Geochemical sediment classification of the Tamatán Group: K_2_O–Na_2_O diagram after [10].

**Fig. 18:**
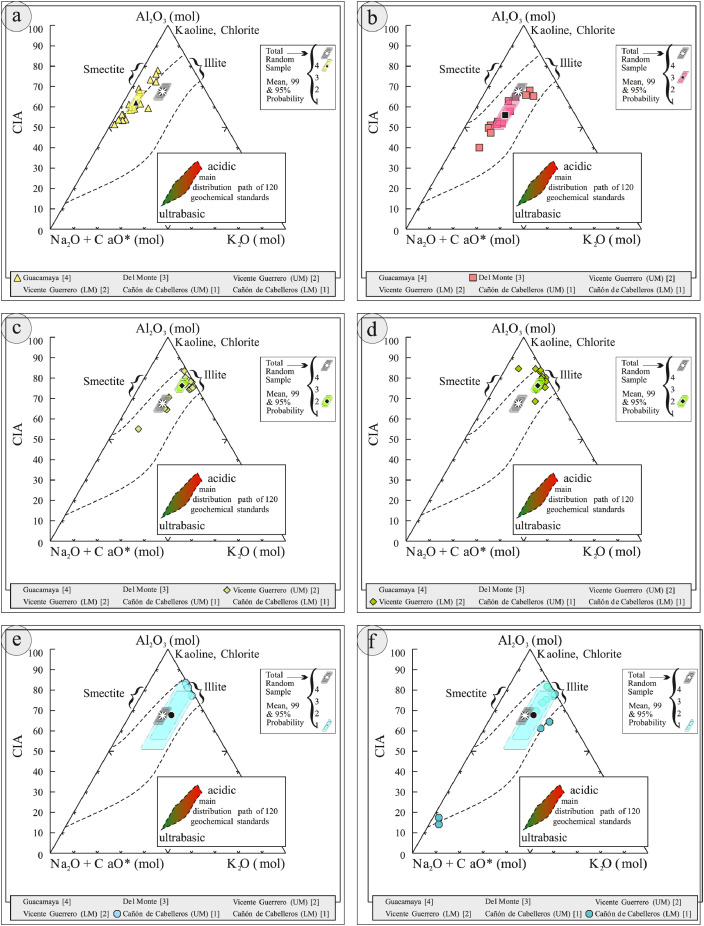
Geochemical Index of Alteration of the sediments of the Tamatán Group: Na_2_O+CaO–Al_2_O_3_–K_2_O diagram after [12,13] modified by [10].

**Fig. 19:**
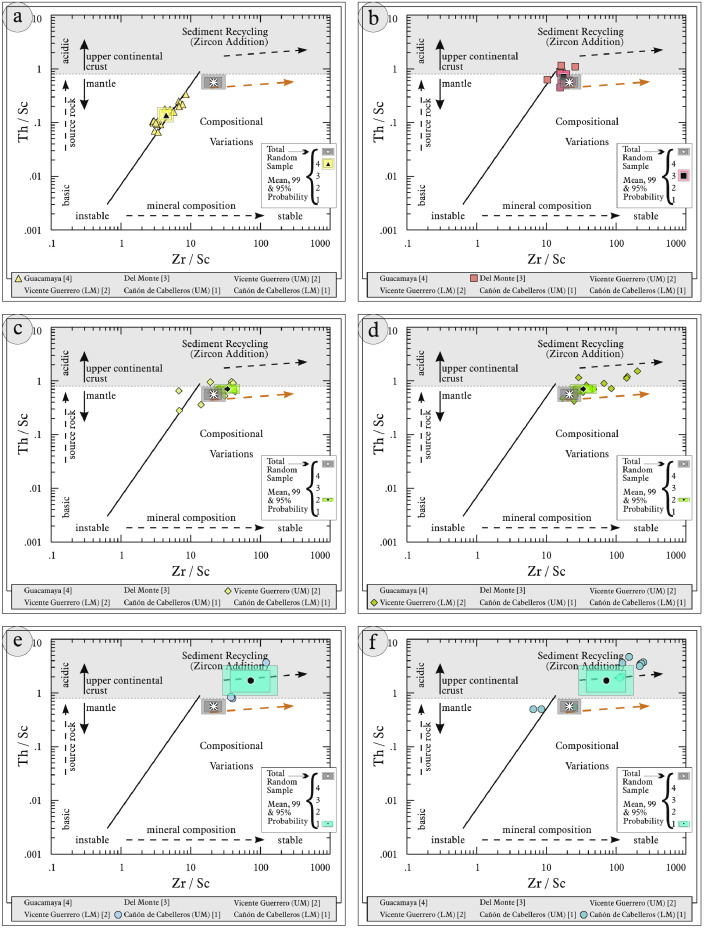
Geochemical classification of recycling of the Tamatán Group: Zr/Sc–Th/Sc diagram after [14].

**Fig. 20:**
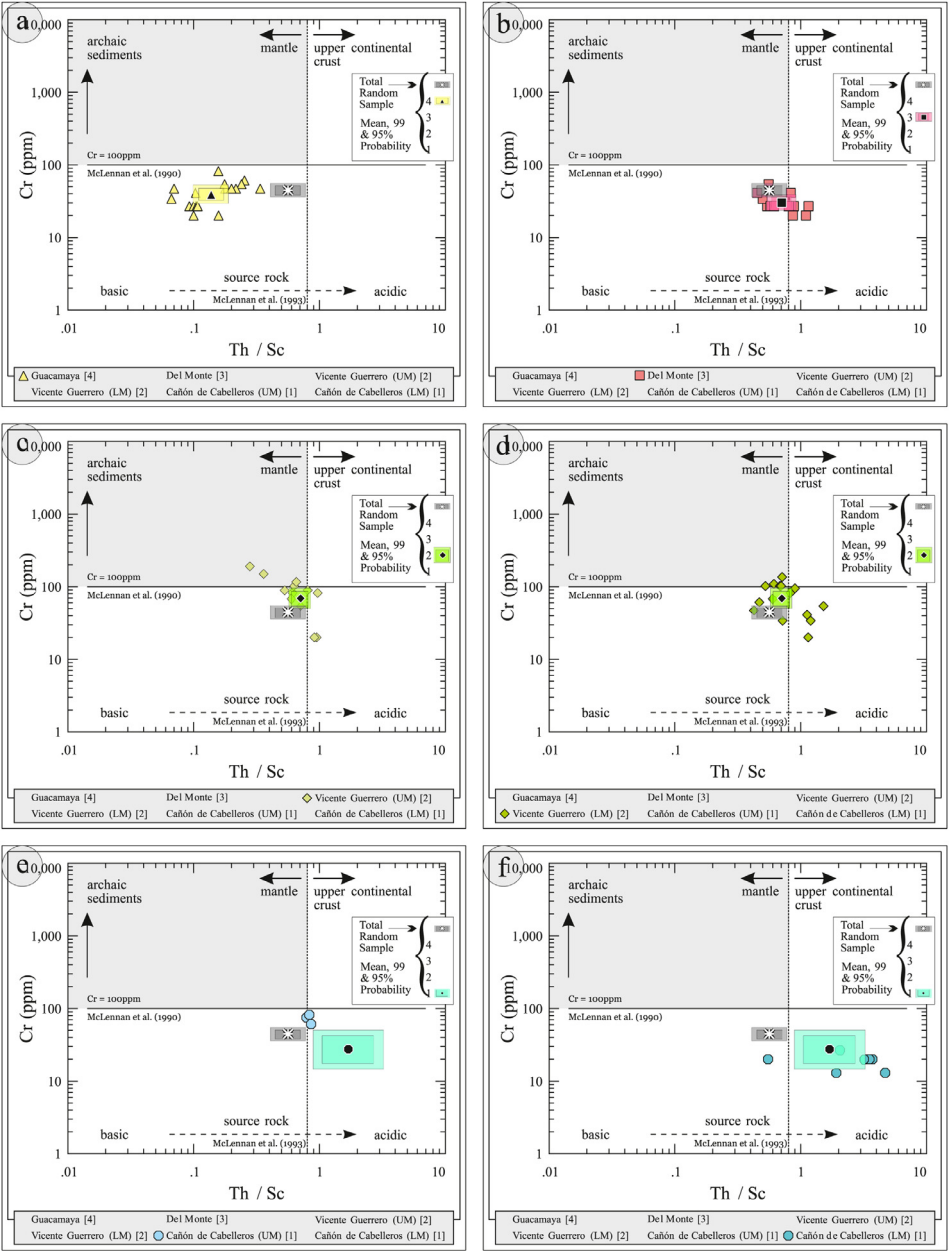
Geochemical classification of the source area and geotectonic environment of the Tamatán Group: Th/Sc–Cr diagram after [10].

**Fig. 21:**
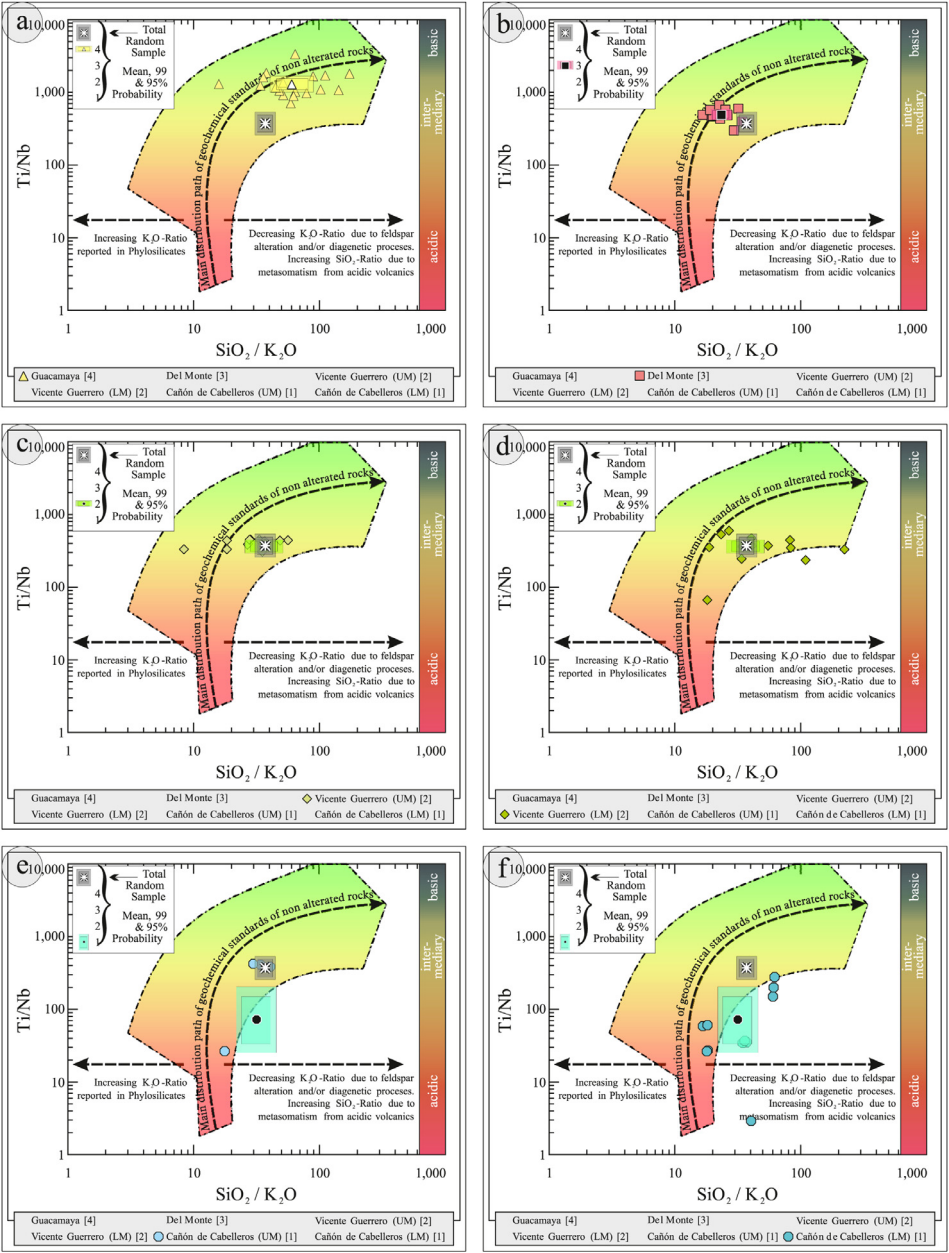
Geochemical classification of the source area of the Tamatán Group: SiO_2_/K_2_O–Ti/Nb diagram after [10].

**Fig. 22:**
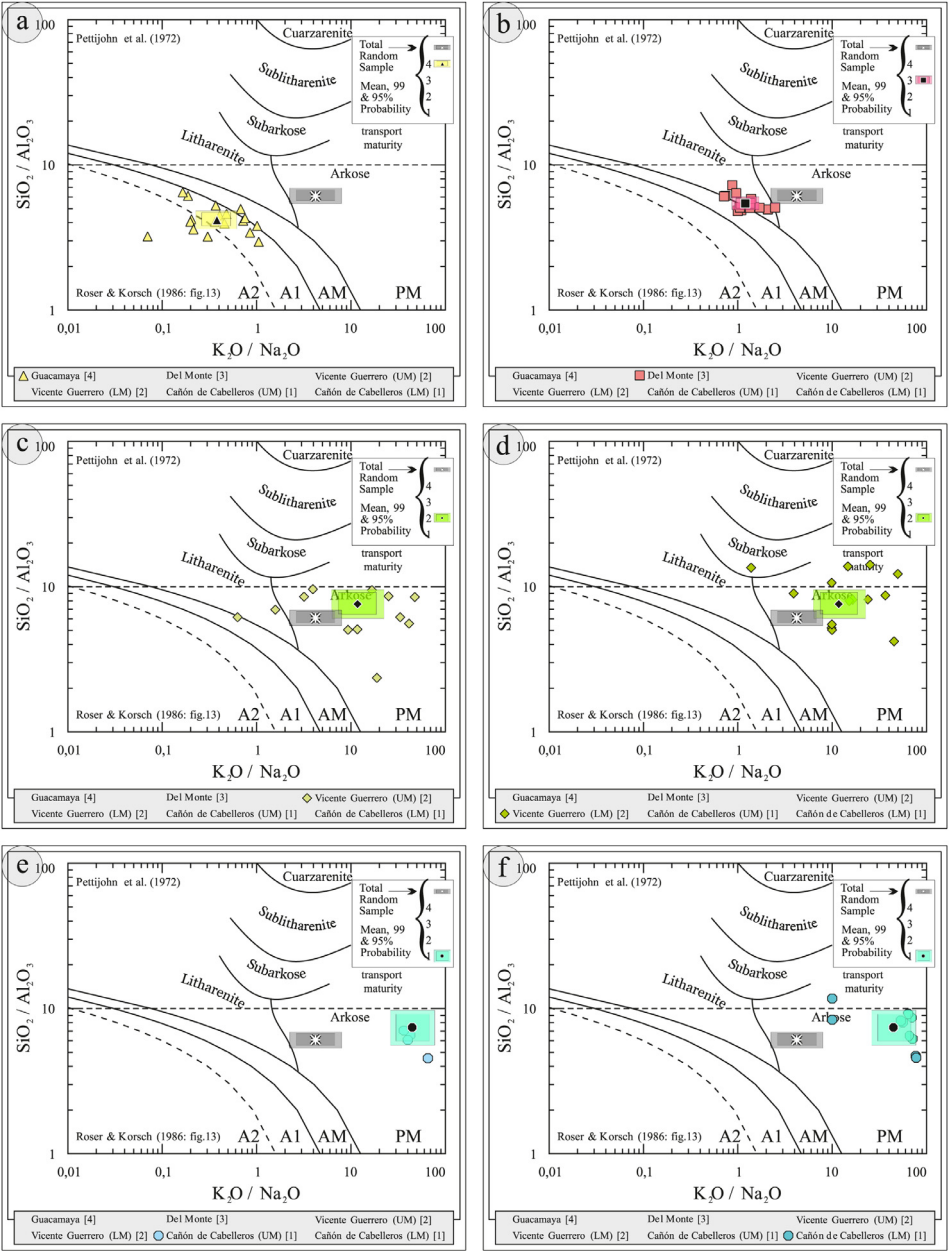
Geochemical sediment classification and classification of the geotectonic environment of the Tamatán Group: K_2_O/Na_2_O–SiO_2_/Al_2_O_3_ diagram after [10].

**Fig. 23:**
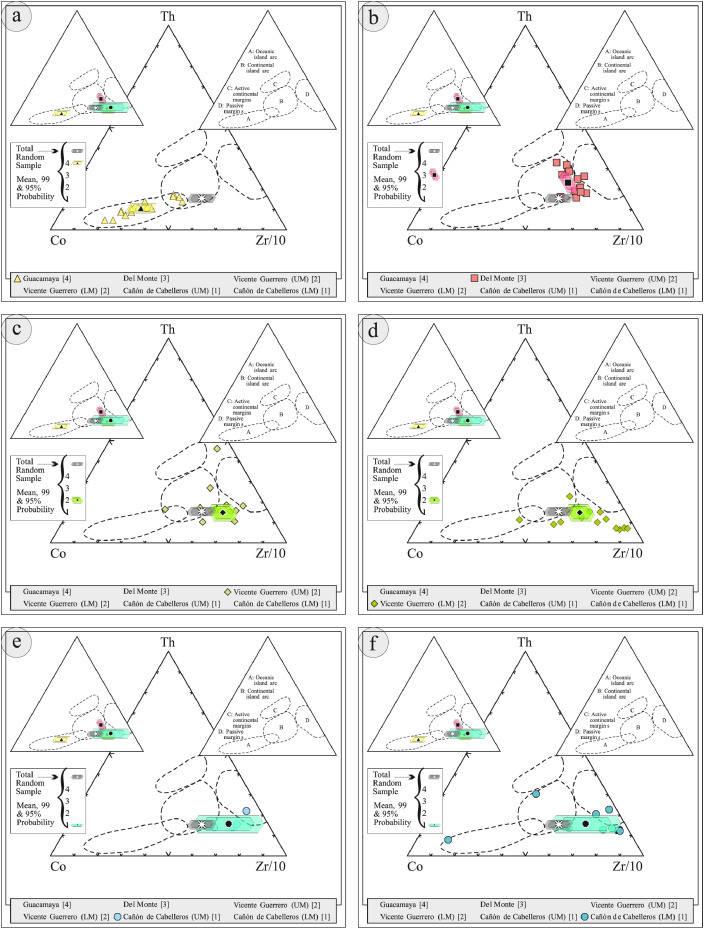
Geochemical classification of the source area and geotectonic environment of the Tamatán Group: Th–Co–Zr/10 diagram after [15].

**Fig. 24:**
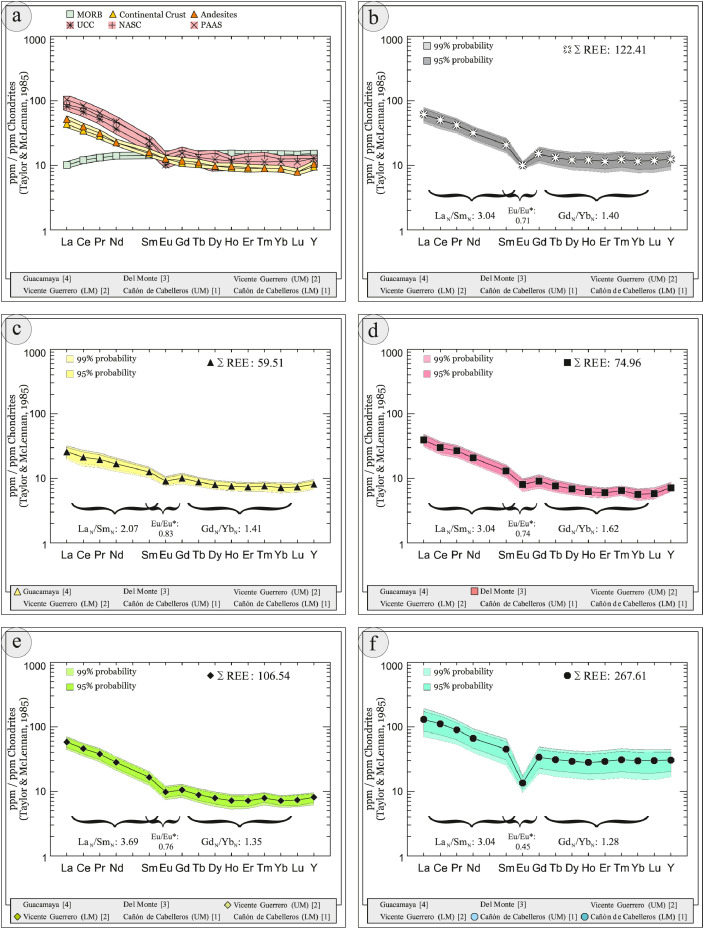
REE pattern of the Tamatán Group after [16].

**Fig. 25:**
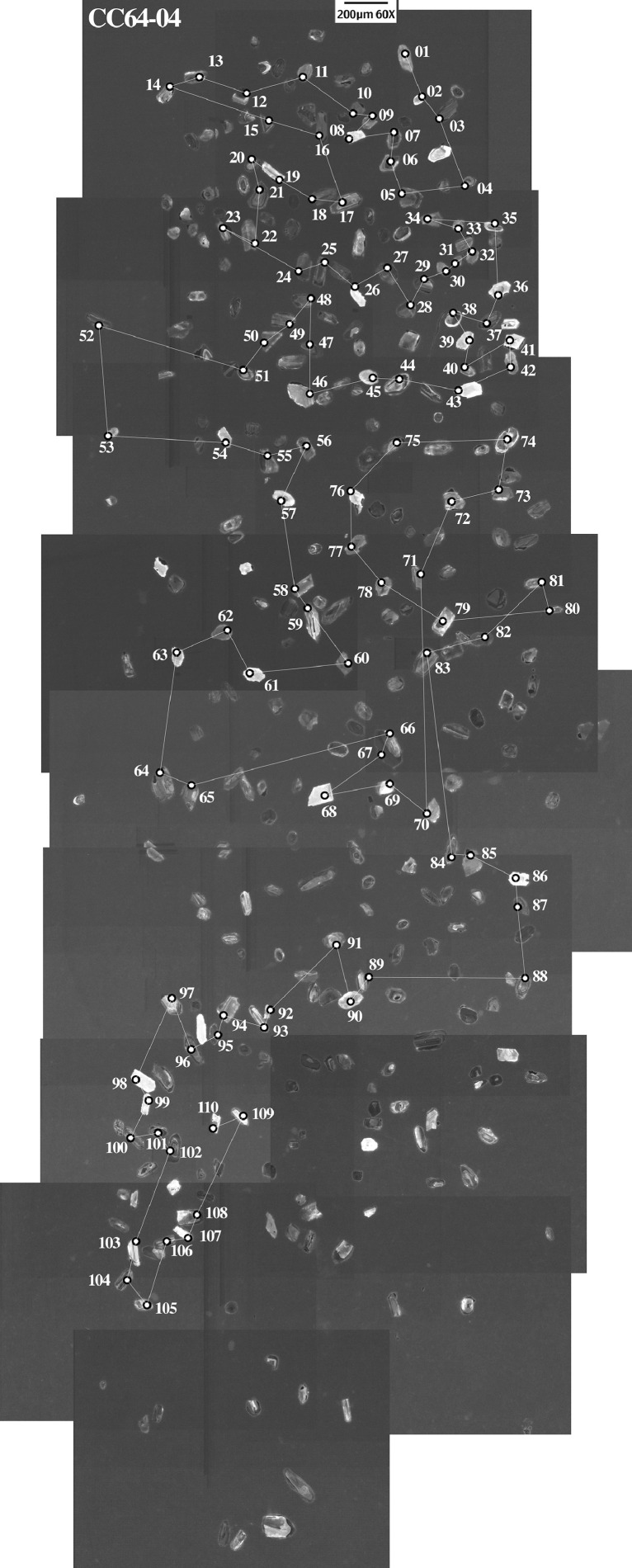
CL-images of detrital zircons from sample CC64–04: Cañón de Caballeros Formation.

**Fig. 26:**
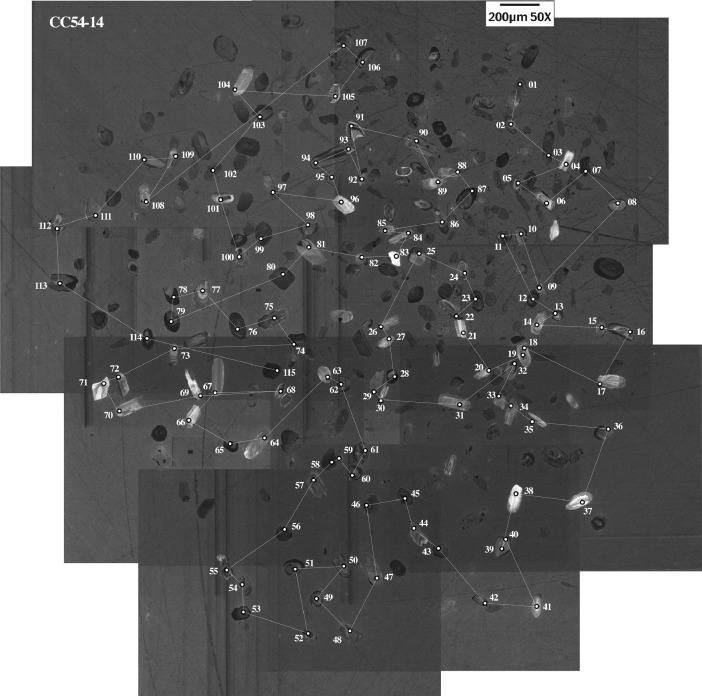
CL-images of detrital zircons from sample CC54–14: Vicente Guerrero Formation.

**Fig. 27:**
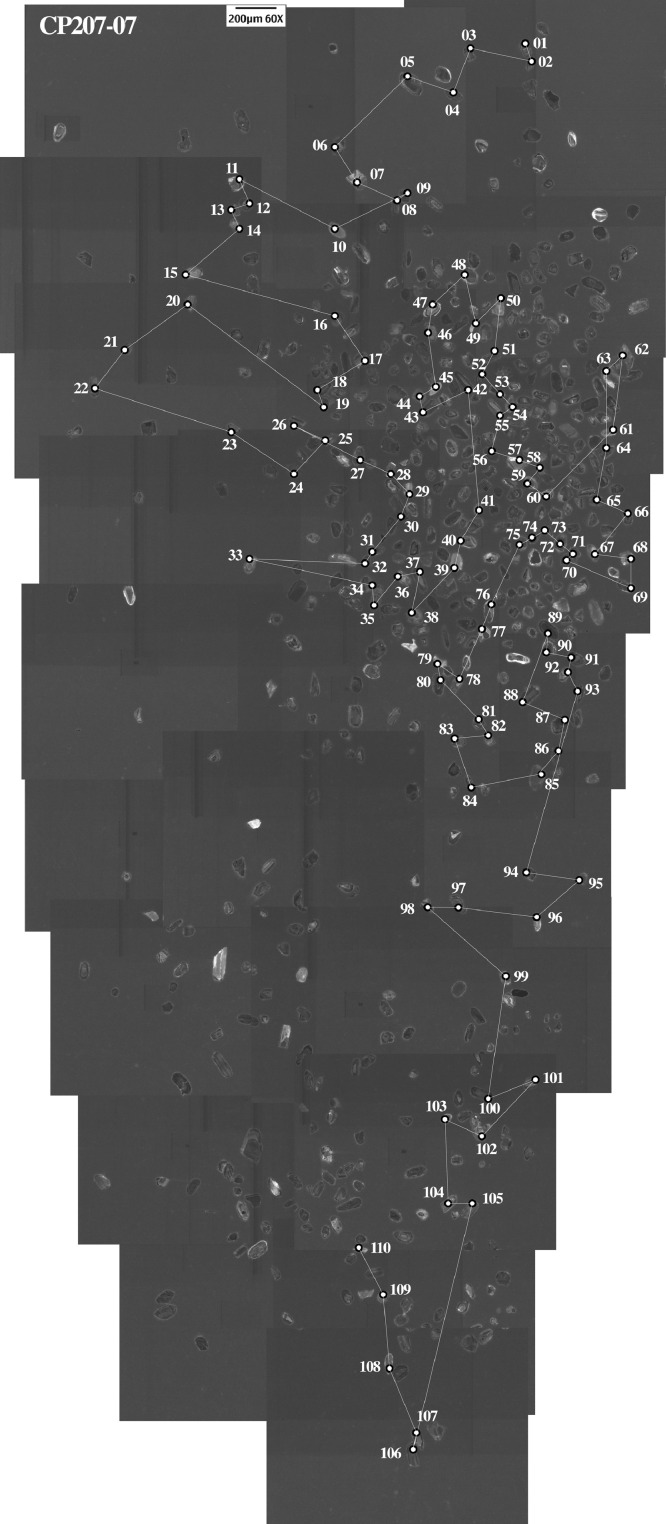
CL-images of detrital zircons from sample C9207–07: Del Monte Formation.

**Fig. 28:**
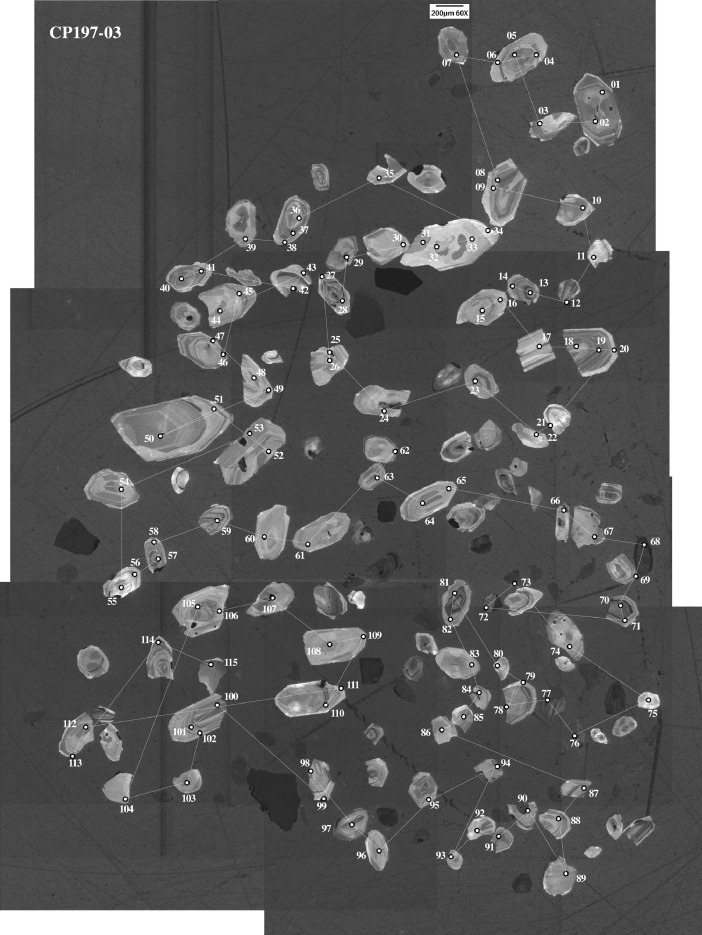
CL-images of detrital zircons from sample CP197–03: Guacamaya Formation.

• Fig. 1: Geological maps of the sampling area to locate the detailed sample collection areas of the different geological units.
• Fig. 2: Location of the collected samples in a stratigraphic column to provide the stratigraphic position of each of the samples.
• Figs. 3–7: Photographs documenting the outcrops of the Cañón de Caballeros Formation (Fig. 3), the Vicente Guerrero Formation (Fig. 4), the Del Monte Formation (Fig. 5), and the Guacamaya Formation (Figs. 6 and 7).
• Table 1: Description of selected samples and its geographical location with coordinates and UTM data. Complete data see Table 1 in [2].
• Table 2: Detailed list of the parameters and characteristics used for statistical point counting of the thin sections.
• Table 3: Raw data of selected point counted thin sections (this table shows the values in counted grains). Complete table see Table 3 in [2].
• Table 4: Recalculated data of selected point counted parameters in thin sections (this table shows the percentage of the total value). Complete data see Table 4 in [2].
• Table 5: Recalculated modal and ternary index data of selected samples. Complete data see Table 5 in [2]. The data for each ternary diagram has to be recalculated to achieve 100% in total. Qt–F–L+Ch after [3]; Qt–F–L tafter [4] and [5]; Qm–F–Lt after [4]; Qm–K–P after [6]; Lm–Lv–Ls and Qp–Lv–Ls after [3] and [5]. Included are the recalculated statistical parameters of the arithmetical mean and the 95% and 99% statistical confidence limits after Student [7].
• Figs. 8–11: Detailed geological map documenting the sample locations of the Cañón de Caballeros Formation (Fig. 8), the Vicente Guerrero Formation (Fig. 9), the Del Monte Formation (Fig. 10), and the Guacamaya Formation (Fig. 11).
• Tables 6–9: Photomicrographs of selected sections from the Cañón de Caballeros Formation (Table 6), the Vicente Guerrero Formation (Table 7), the Del Monte Formation (Table 8), and the Guacamaya Formation (Table 9). On the left side a standard magnification with parallel and crossed nicols and on the right side detail enlargements from the same perspective, also with parallel and crossed nicols. Complete data see Tables 6–9 in [2].
• Fig. 12: Recalculated petrographic point counting data, mean-, and confidential limits plotted, separated for each stratigraphic unit, into the Q–F–L diagram after [3].
• The Figs. 12–15 contain recalculated petrographic point counting data, mean-, and confidential limits plotted, separated for each stratigraphic unit, into the Q–F–L diagram after [8] (Fig. 12), Qm–P–K diagram after [6] (Fig. 13), the Q–F–L diagram after [4] (Fig. 14), and the Qm–F–Lt diagram after [9] (Fig. 15).
• The Tables 10 and 11 contain geochemical raw data and selected (recalculated) geochemical parameters of selected samples from the Cañon de Caballeros Formation and the Vicente Guerrero Formation (Table 10), and, from the Del Monte Formation and the Guacamaya Formation (Table 11). Note: Oxides and LOI in%, other elements in ppm. Abbreviations: n.d.: not detected; CaCO* = maximum CaO in Carbonates recalculated from CO_2_; Chem.Lit: Chemical lithology [10]; CaO++: CaO enriched samples; Psam.: Psammite classified samples; Rest++: enriched in Si_O2_ and Al2O_3_; Rest–: impoverished in SiO_2_ and Al_2_O_3_; Eu/Eu*=Eu_N_/(Sm_N_xGd_N_)0.5; REE= La_N_/Lu_N_; LREE=La_N_/Sm_N_; HREE=Gd_N_/Lu_N_; ∑-REE= Total REE (in ppm); Samples are not LOI-free recalculated. Complete data see Tables 10–13 in [2].
• Table 12 (Table 14 in [2]): Simple statistics of the selected geochemical parameters which are used in the discrimination diagrams. Included are the recalculated statistical parameters of the arithmetical mean and the 95% and 99% statistical confidence limits after Student [7].
• Table 13 (Table 15 in [2]): Simple statistics of the rare earth elements (REE). Included are the recalculated statistical parameters of the arithmetical mean and the 95% and 99% statistical confidence limits after Student [7].
• The Figs. 16–24 contain data from sedimentary whole rock geochemical analysis (mean-, and confidential limits are recalculated) plotted, separated for each stratigraphic unit, plotted into the SiO_2_–Al_2_O_3_ diagram after [10] (Fig. 16), the K_2_O–Na_2_O diagram after [11]. (Fig. 17), the Na_2_O+CaO–Al_2_O_3_–K_2_O diagram after [12,13] modified by [10] (Fig. 18); the Zr/Sc–Th/Sc diagram after [14] (Fig. 19), the Th/Sc–Cr diagram after [10] (Fig. 20), the SiO_2_/K_2_O–Ti/Nb diagram after [10] (Fig. 21), the K_2_O/Na_2_O–SiO_2_/Al_2_O3 diagram after [10] (Fig. 22), the Th–Co–Zr/10 diagram after [15] (Fig. 23), and the REE-Spider diagram after [16] (Fig. 24).
• The Tables 14, 16, 18, and 20 of this paper contain LA-MC-ICPMS U-Th-Pb raw and processed data of selected detrital zircons from Cañón de Caballeros Formation (Table 14), Vicente Guerrero Formation (Table 16) Del Monte Formation (Table 18), and the Guacamaya Formation (Table 20). Complete data see Tables 16, 18, 20, and 22 in [2].
• The Figs. 25–28 show unfiltered CL images (raw images) of the collection of zircons from sample CC64–04 (Cañón de Caballeros Formation; Fig. 25), sample CC54–14 (Vicente Guerrero Formation; Fig. 26), sample C9207–07 (Del Monte Formation; Fig. 27), and sample CP197–03 (Guacamaya Formation; Fig. 28). See also Figs. 46–49 in [2].
• The Tables 15, 17,19, and 21 of this paper contain single grain CL-images of selected measured zircons of the Cañón de Caballeros Formation (Table 15), the Vicente Guerrero Formation (Table 17), the Del Monte Formation (Table 19), and the Guacamaya Formation (Table 21), amplified from the figures Figs. 25–28 (complete data see [2]). The images are arranged in descending order of the best ages. The colors of the boxes correspond to the colors of the International Chronostratigraphic Table of the respective System/Period (for Precambrian ages) or Series/Epoch (for Paleozoic ages). Complete data see Tables 17, 19, 21, and 23 in [2].
• Tables 24–39 in [2] show the single grain CL-images of selected measured zircon of the Siderian (Table 24), Statherian (Table 25), Calymmian (Table 26), Ectasian (Table 27), Stenian (Table 28), Tonian (Table 29), Cryogenian (Table 30), Ediacaran (Table 31), Cambrian (Table 32), Ordovician (Table 33), Silurian (Table 34), Devonian (Table 35), Mississippian (Table 36), Pennsylvanian (Table 37), Permian (Table 38), and Triassic (Table 39) ages, amplified from the Figs. 46–49. The images are arranged in descending order of the best ages. The colors of the boxes correspond to the color of the respective stratigraphic unit as used in the whole text (blue: Cañón de Caballeros Fm.; green: Vicente Guerro Fm.; red: Del Monte Fm.; and yellow: Guacamaya Fm.).

## 2 Supplemantary Data Set

The supplementary data set provides the complete data from 105 samples. From 70 samples, photomicrographs were taken and point-counted and modal analyses on recalculated petrographic parameters were provided. Four samples for U–Pb geochronological zircon analyses were made. The sample location is given with the geographical and UTM coordinates of each sample. Each sample is located on a geological map.Outcrop photographs are provided. Geochemical analyses (major, trace, and rare earth elements [REE]) of 73 samples were conducted. The petrographic and geochemical data are presented as raw data and displayed as a simple statistic of the selected petrography and geochemical parameters, respectively. Petrographical and geochemical compositional data and its arithmetic mean and confidence limits (95% and 99%, respectively) are plotted into the most common diagrams offered by several authors. The supplementary data set contains single zircon CL-images and best ages of each zircon (< 400 grains) of the Cañón de Caballeros Formation, the Vicente Guerrero Formation, the Del Monte Formation, and the Guacamaya Formation. Also single grain CL-images of each measured zircon of the Siderian, Statherian, Calymmian, Ectasian, Stenian, Tonian, Cryogenian, Ediacaran, Cambrian, Ordovician, Silurian, Devonian, Mississippian, Pennsylvanian, Permian, and Triassicages, amplified from the Figs. 46–49. The images are arranged in descending order of the best ages.

## 3 Experimental Design, Materials and Methods

The cartographic basis for the fieldwork comprised topographical maps of the Instituto Nacional de Estadística y Geografía (INEGI) on a scale of 1:10,000 and is based on geological maps proposed by Stewart et al. [17] and [18]. The outcrops (Fig. 1) and sample sites were located on the geological map. A detailed description of the sampling and sample processing is given in Table 1.

Thin sections, documented in Table 6, Table 7, Table 8, Table 9, are characterized and photographed using a Leica MC170HD polarization microscope and a HC FL PLAN 2.5 × 0.07 camera under parallel and crossed Nicols conditions. According to the Gazzi–Dickinson technique to minimize the effects of the size of the clasts, modal analyses were performed on 71 samples by counting 400–600 points. The modal composition and statistical parameters of the point counting were based on previous works [3,5,6,4,9,8]. The 95% and 99% confidence intervals for Student's *t*-test [7] were plotted in optically distinct shades (see Fig. 12, Fig. 13, Fig. 14, Fig. 15, Fig. 16, Fig. 17, Fig. 18, Fig. 19, Fig. 20; Table 2, Table 3, Table 4, Table 5).

The analyses were performed using inductively coupled plasma optical emission spectrometry for major elements and inductively coupled plasma mass spectrometry for trace elements at ACME Analytical Laboratories Ltd. in Vancouver, Canada. The analyses are provided in Table 9. For this data collection, the distributions of the elements in the random samples were described using the arithmetic mean and confidence limits (95% and 99%, respectively) supplied by Student's *t*-test ([7]).

Four samples underwent U–Pb geochronological analysis. A detailed description of the geochronological processing and analytic methods is given [1]. The raw and processed data of selected samples are listed in Tables 14, 16, 18, and 20. The four samples were processed following standard procedures at the geology department of Centro de Investigación Científica y de Educación Superior de Ensenada Baja California. U–Pb data were analyzed in zircon grains using laser ablation multicollector inductively coupled plasma mass spectrometry at the Central Analytical Facilities, Stellenbosch University, South Africa. All the geochronological data were plotted into the Wetherill Concordia and relative age probability diagram [19].

## 4 Figures and Tables

### 4.1 Geological setting and sampling

Fig. 1, Fig. 2, Fig. 3, Fig. 4, Fig. 5, Fig. 6, Fig. 7, Table 1.

**Table 1:** Sample list and sample locations of selected samples (complete list see [2]).

								Analytical Method		
Sample	Lithology	Canyon	Formation	UTM-E	UTM-N	Latitude (°N)	Longitude (°W)	I	II	III
CC64–01	MeS	Caballeros	Cañón de Caballeros (Lm)	470,094	2,631,870	23°47′52.03′′	99°17′36.85′′	**X**	**X**	
CC64–02a	Sh	Caballeros	Cañón de Caballeros (Um)	470,153	2,631,822	23°47′50.47′′	99°17′34.76′′	**X**	**X**	
CC64–02b	Sh	Caballeros	Cañón de Caballeros (Um)	470,153	2,631,822	23°47′50.47′′	99°17′34.76′′	**X**	**X**	
CC64–03a	CS	Caballeros	Cañón de Caballeros (Lm)	470,276	2,631,703	23°47′46.61′′	99°17′30.41′′	**X**	**X**	
CC64–03b	CS	Caballeros	Cañón de Caballeros (Lm)	470,276	2,631,703	23°47′46.61′′	99°17′30.41′′	**X**	**X**	
CC64–04	CS	Caballeros	Cañón de Caballeros (Lm)	470,240	2,631,770	23°47′48.79′′	99°17′31.69′′	**X**	**X**	**X**
EMA1–04	Cgl	Peregrina	Vicente Guerrero (Um)	472,991	2,629,012	23°46′19.28′′	99°15′54.29′′	**X**	**X**	
EMA1–05	MeS	Peregrina	Vicente Guerrero (Um)	472,991	2,629,012	23°46′19.28′′	99°15′54.29′′	**X**	**X**	
EMA1–07	FS	Peregrina	Vicente Guerrero (Um)	472,889	2,629,075	23°46′21.32′′	99°15′57.89′′	**X**	**X**	
EMA2–10	MeS	Caballeros	Vicente Guerrero (Lm)	469,794	2,632,328	23°48′06.90′′	99°17′47.49′′	**X**	**X**	
CC54–14	MeS	Caballeros	Vicente Guerrero (Lm)	469,772	2,632,328	23°48′06.90′′	99°17′47.49′′	**X**	**X**	**X**
CC54–15	MeS	Caballeros	Vicente Guerrero (Lm)	469,772	2,632,328	23°48′06.90′′	99°17′47.49′′	**X**	**X**	
EMA1–01	MeS	Peregrina	Del Monte	473,140	2,629,008	23°46′19.16′′	99°15′49.02′′	**X**	**X**	
EMA1–02	MeS	Peregrina	Del Monte	473,072	2,629,012	23°46′19.29′′	99°15′51.42′′	**X**	**X**	
EMA2–06	FS	Caballeros	Del Monte	470,841	2,633,811	23°48′55.19′′	99°17′10.59′′	**X**	**X**	
EMA2–07	CS	Caballeros	Del Monte	470,841	2,633,811	23°48′55.19′′	99°17′10.59′′	**X**	**X**	
EMA2–09	Cgl	Caballeros	Del Monte	470,915	2,633,706	23°48′51.79′′	99°17′07.97′′	**X**	**X**	
CP207–07	MeS	Peregrina	Del Monte	473,181	2,629,063	23°46′20.95′′	99°15′47.58′′	**X**	**X**	**X**
EMA1–14	CS	Peregrina	Guacamaya	474,556	2,625,094	23°46′22.05′′	99°14′59.00′′	**X**	**X**	
EMA1–15	CS	Peregrina	Guacamaya	474,833	2,629,133	23°46′23.32′′	99°14′49.21′′	**X**	**X**	
EMA2–01	Cgl	Caballeros	Guacamaya	471,266	2,633,885	23°48′57.63′′	23°16′55.58′′	**X**	**X**	
EMA2–02	Cgl	Caballeros	Guacamaya	471,033	2,634,126	23°48′05.45′′	99°17′03.83′′	**X**	**X**	
CC34–08	Cgl	Caballeros	Guacamaya	471,235	2,633,893	23°48′57.88′′	99°16′56.67′′	**X**	**X**	
CP197–03	CS	Peregrina	Guacamaya	474,593	2,629,044	23°46′20.42′′	99°14′57.69′′	**X**	**X**	**X**

**Abreviations**: Sh= Shale, FS= Fine sandstone, MeS = Medium sandstone, CS= Coarse sandstone, Cgl = Conglomerate, Lm= Lower member, Um= Upper member. Methods of analyses: Pet.= Petrography, Gq = Geochemical and Gch = Geochronology.

### 4.2 Petrographical data

Table 2, Table 3, Table 4, Table 5, Table 6, Table 7, Table 8, Table 9, Fig. 8, Fig. 9, Fig. 10, Fig. 11, Fig. 12, Fig. 13, Fig. 14, Fig. 15.

**Table 2:** Point counting parameters.

Counted	Grains/Fragments	Parameter	Calculation	Meaning
Qmr*	straight extinction monocrystalline quartz	Qm	Qm+Qmr+Cq	Monocrystalline quartz
Qmo*	undulatory monocrystalline quartz			
Qp2–3*	polycrystalline quartz with 2–3 units	Qp	Qp2–3+Qp< 3	Polycrystalline quartz
Qp>3*	polycrystalline quartz with >3 units			
Cq*	quartz with calcite and/or illite			

		Qt	Qm+Qp	Total stable quartz
Fk*	potassic feldspar without replacement	K	Fk+Fki+Frc	Potassic feldspar
Fki*	potassic feldspar with illite replacement			
Frc*	potassic feldspar with calcite replacement			
Pna*	sodic plagioclase without replacement	P	Pna+Pki+Prc	Plagioclase
Pki*	sodic plagioclase with illite/sericite replacement			
Prc*	sodic plagioclase with calcite replacement			

		F	K + P	Total feldspars
Lmf*	psammitic lithic	Lm	Lmf + Lmp	Metamorphic lithics
Lmp*	metapelitic lithic			
Lvf*	felsitic volcanic lithic,	Lv	Lvf+Lvt+Lvl+Lp	Igneous lithics
Lvt*	tobaceous volcanic lithic,			
Lvl*	lathwork volcanic lithic			
Lp*	plutonic lithic			
Lsa*	sandy sedimentary lithic	Ls	Lsa+Lslu+LsCe+LsCm+Fo+LsBi	Sedimentary lithics
Lslu*	shale/limolite sedimentary lithic			
LsCe**	sparitic sedimentary lithic			
LsCm**	micritic sedimentary lithic			
Fo**	microfossils			
LsBi***	bioclastic sedimentary lithic			
Ch	chert			

		Lt	Lm+Lv+Ls	Total unstable lithic
M	mica (include biotite;Bt, muscovite; Ms and/or chlorite; Chl)			
Hm	heavy mineral (include: zircon; Zr and/or apatite; Ap)			

**Abreviations**: Cm = Calcitic cement / clay matrix, Sor. = sorted, P- M- W = poorly, moderate and well sorted, Ro. = roundness, Sub-a = sub-angular grains, Sub-r = sub-rounded grains, r = rounded grains, Ma = mature, SubMa = submature, InMa = innmature. * = Noncarbonate extrabasinal grains (NCE), ** = Carbonatic extrabasinal components (CE), *** = Carbonatic intrabasinal components (CI). The mainly parameters are in bold**.**

**Table 3:** Point-counting data.

**Table 4:** Percentage of the point-counting data.

**Table 5:** Recalculated modal and ternary index data.

**Table 6:** Photmicrographies of thin sections from the Cañón de Caballeros formation (Upper Silurian-Lower Devonian).

**Table 7:** Photmicrographies of thin sections from the Vicente Guerrero formation (Mississippian).

**Table 8:** Photmicrographies of thin sections from the Del Monte formation (Pennsylvanian).

**Table 9:** Photmicrographies of thin sections from the Guacamaya formation (Permian).

### 4.3 Geochemical data

Table 10, Table 11, Table 12, Table 13, Fig. 16, Fig. 17, Fig. 18, Fig. 19, Fig. 20, Fig. 21, Fig. 22, Fig. 23, Fig. 24.

**Table 10:** Geochemical parameters of selected samples from the Cañon de Caballeros and Vicente Guerrero formations.

**Table 11:** Geochemical parameters of selected samples from the Vicente Guerrero and Del Monte formations.

**Table 12:** Simple statistics of the selected geochemical parameters of the Tamatán Group.

**Table 13:** Simple statistics of the rare earth elements (REE) of the Tamatán Group.

### 4.4 U-Th-Pb data of detrital zircons

Table 14, Table 15, Table 16, Table 17, Table 18, Table 19, Table 20, Table 21.

**Table 14:** LA-MC-ICPMS U-Th-Pb data of detrital zircons from the Cañón de Caballeros Formation: Sample CC64–04, Caballeros Canyon, Huizachal-Peregrina Anticlinorium, Tamaulipas, México; Coordinates: R 14–470,240 E, 2,631,770 N.

**Table 15:** CL-images and best ages from selected zircons of the Cañón de Caballeros Formation (Silurian).

**Table 16:** LA-MC-ICPMS U-Th-Pb data of selected detrital zircons from the Vicente Guerrero Formation: Sample CC54–14, Caballeros Canyon, Huizachal-Peregrina Anticlinorium, Tamaulipas, México; Coordinates: R 14–4,697,720 E, 2,632,328 N.

**Table 17:** CL-images and best ages from zircons of the Vicente Guerrero Formation (Mississippian).

**Table 18:** LA-MC-ICPMS U-Th-Pb data of detrital zircons from the Del Monte Formation: Sample CC207–07, Peregrina Canyon, Huizachal-Peregrina Anticlinorium, Tamaulipas, México; Coordinates: R 14–473,181 E, 2,629,063 N.

**Table 19:** CL-images and best ages from zircons of the Del Monte Formation (Pennsylvanian).

**Table 20:** LA-MC-ICPMS U-Th-Pb data of detrital zircons from the Guacamaya Formation: Sample CC197–03, Peregrina Canyon, Huizachal-Peregrina Anticlinorium, Tamaulipas, México; Coordinates: R 14–474,593 E, 2,629,044 N.

**Table 21:** CL-images and best ages from zircons of the Guacamaya Formation (Permian).

## Ethics Statement

• This material is the authors' own original work, which has not been previously published elsewhere.
• The paper is not currently being considered for publication elsewhere.
• The paper reflects the authors' own research and analysis in a truthful and complete manner.
• The paper properly credits the meaningful contributions of co-authors and co-researchers.
• The results are appropriately placed in the context of prior and existing research.
• All sources used are properly disclosed (correct citation).
• All authors have been personally and actively involved in substantial work leading to the paper, and will take public responsibility for its content.
• No information obtained for experimentation with human subjects was used.
• No information obtained for experimentation with animals was used.

## CRediT Author Statement

**Juan Moisés Casas-Peña**: Cartography; Data collection; Data curation (petrology, geochemistry); Writing; **Juan Alonso Ramírez-Fernández**: Conceptualization; Methodology; Project administration; Data curation (petrology); **Fernando Velasco-Tapia**: Data curation (geochemistry); **Eduardo Alejandro Alemán-Gallardo**: Cartography; Data collection; **Carita Augustsson**: Conceptualization; Data collection; Data curation (geochemistry); **Bodo Weber**: Data curation (U-Pb); **Dirk Frei**: Data curation (U-Pb); **Uwe Jenchen**: Conceptualization; Methodology; Writing-Reviewing & Editing; Project administration; Fundraising.

## Declaration of Competing Interest

The authors declare that they have no known competing financial interests or personal relationships which have or could be perceived to have influenced the work reported in this article.
